# Adsorption Behavior of Chiral Pharmaceuticals onto Montmorillonite Clay: Evaluating Removal Efficiency and Stereoselectivity

**DOI:** 10.3390/molecules31122040

**Published:** 2026-06-11

**Authors:** Gül Gülenay Hacıosmanoğlu, Marina Arenas, Carmen Mejías, Julia Martín, Juan Luis Santos, Irene Aparicio, Esteban Alonso

**Affiliations:** 1 Environmental Engineering Department, Faculty of Engineering, Marmara University, Uyanık Cd. No. 6, 34840 Istanbul, Turkey; 2 Departamento de Química Analítica, Escuela Politécnica Superior, Universidad de Sevilla, C/Virgen de África 7, E-41011 Sevilla, Spain; mamolina@us.es (M.A.); cmpadilla@us.es (C.M.); jlsantos@us.es (J.L.S.); iaparicio@us.es (I.A.); ealonso@us.es (E.A.)

**Keywords:** chiral pharmaceuticals, enantiomers, montmorillonite, adsorption, water, wastewater

## Abstract

Chiral pharmaceuticals (CPs) have gained growing attention in environmental studies regarding the differential behavior of individual enantiomers in racemic mixtures. This study investigates the stereoselectivity and efficiency of montmorillonite (MMT), a natural and low-cost adsorbent, for the removal of a wide group chiral pharmaceuticals and metabolites (atenolol, propranolol, metoprolol, fluoxetine, venlafaxine, norfluoxetine, and O-desmethylvenlafaxine). The effects of adsorption conditions including initial CP concentration, contact time, adsorbent dose, solution pH, and humic acid content were evaluated. In most adsorption experiments, no significant stereoselective behavior was observed, except for the case where a low adsorbent dose was applied. Interestingly, as the solution humic acid content increased (up to 40 mg/L), the adsorption capacity was increased for most of the target CPs. Isotherm studies revealed that the Freundlich model described the experimental data well and the process was favorable. Adsorption mechanism was interpreted by material characterization before and after adsorption. High removal efficiencies (88.0 to 99.8%) and the non-enantioselective behavior of MMT indicate that it can be used effectively for the simultaneous removal of both enantiomeric forms of various chiral pharmaceuticals from aqueous matrices.

## 1. Introduction

Chiral pharmaceuticals (CPs) represent a significant group of prescription drugs widely used in human and veterinary medicine. Enantiomers of CPs have the same chemical structures, except they are non-superimposable on their mirror images. These compounds have received great scientific interest since the enantiomers of the same chiral pharmaceuticals can display different pharmacological activities and toxicities. Especially after the recognition of radically different biological activities of enantiomeric compounds, including therapeutic effects and severe side effects (e.g., toxicity, mutagenicity, teratogenicity), strict regulations were introduced to test the enantiomers [1], while analytical method development for the recognition, separation, and identification of enantiomers gained attention.

From an environmental point of view, CPs are constantly released into wastewater streams via human and animal consumption. It was shown that conventional wastewater treatment processes are not capable of completely eliminating pharmaceutical residues, including chiral compounds [2,3,4,5]. As a result, these compounds find their way into different environmental compartments. CPs in the effluent streams are usually detected in ng/L to μg/L concentration ranges [6]. In addition to the discharge via effluent streams, the possible complex interactions in the environment such as bioaccumulation, biomagnification, and synergistic activity with different contaminants can aggravate the problem [7,8,9].

Once released into the environment, the fate and behavior of enantiomers of CPs depend on diverse factors, such as the properties of the chiral pollutant, environmental matrix, treatment technology used; activity of the microbial communities; and spatial and temporal environmental factors [10]. Studies have shown that chiral compounds can exhibit enantioselective behavior in different environmental matrices [11,12,13]. Regarding differential toxicity and environmental fate of the enantiomers of chiral contaminants, studies on developing effective treatment strategies toward both enantiomeric forms of CPs are of crucial importance. However, there are limited studies on this topic, mainly because the enantioselective analysis of CPs in the environmental samples represents considerable problems and challenges. These problems and difficulties were described in detail in recent reviews [14,15]. Moreover, there is poor experimental evidence of stereoselective adsorption of CPs, probably because, as an abiotic process, it is considered non-stereoselective. However, solid environmental matrices (organic matter, minerals, and others) may behave as chiral environments where the enantiomer can exhibit a different behavior [10,14].

This study evaluated the simultaneous adsorption of several different CPs by montmorillonite (MMT). MMT was selected as the adsorbent material because it is a highly abundant, eco-friendly, natural, and inexpensive material. As a 2:1 layered phyllosilicate clay mineral, MMT was shown to be effective for the adsorption of many emerging contaminants. Recent studies on clay-based adsorption of emerging contaminants include paracetamol and metformin [16], ciprofloxacin [17], 2,4-dichlorophenol [18], per- and polyfluoroalkyl substances [19], safinamide [20], propranolol [21], and carbamazepine [22].

In the literature, there are studies on the adsorption of enantiomeric compounds onto different materials for analytical separation [23], detection [24], and biomedical [25] applications. However, this study pioneers the investigation of the adsorptive removal of enantiomeric compounds for environmental remediation purposes. To this end, systematic adsorption studies were conducted and the adsorption behaviors of enantiomer pairs were compared. Moving beyond conventional single-pollutant models, this research evaluates a complex, multi-component mixture of target pollutants to simulate environmentally relevant conditions. The selected model compounds included chiral beta-blockers (atenolol, propranolol, metoprolol) and antidepressants (fluoxetine and venlafaxine), along with their metabolites (norfluoxetine and O-desmethylvenlafaxine). The practical applicability was tested using real-world aquatic samples (i.e., wastewater, tap water, and surface water). Finally, beyond empirical observations, this work focuses on the physical and chemical adsorption mechanisms driving the process.

## 2. Results and Discussion

### 2.1. Characterization of the Adsorbent

SEM analysis was used to visualize the morphological characteristics of MMT. The results are shown in Figure S1 (Supplementary Material). For MMT before adsorption, it can be observed that the surface has an irregular and porous structure. Moreover, Figure S1 indicates that MMT samples consist of mesopores and macropores regarding the pore size classification, i.e., micropores (pores of width ≤ 2 nm), mesopores (pores of a range width of 2–50 nm), and macropores (pores of width > 50 nm) [26]. This observation was supported by the nitrogen adsorption–desorption results (Figure S2). The mean pore size of MMT calculated by Barrett–Joyner–Halenda analysis was 6.76 nm, corresponding to macroporous structure. Based on the Brunauer–Emmett–Teller method, the specific surface area (SSA) of MMT was about 221 m^2^/g.

The thermogravimetric (TGA) analysis results for MMT are given in Figure S3. The overall weight loss during the process was measured as 8.93%. In Figure S3, it can be seen that two distinct thermal degradation events on MMT occur, which indicate the evaporation of physically adsorbed water (<100 °C) and dihydroxylation of clay layers (>500 °C) [27].

Zeta potential analyses were conducted for different pH conditions (in the range of 2–11) and the obtained data are presented in Figure S4. These results show a negative zeta potential of MMT for the studied pH range, indicating that MMT’s surface is negatively charged. This can be explained by the isomorphic substitution of Si^4+^ by Al^3+^ and Fe^3+^ ions in the tetrahedral sheets, and Al^3+^ and Fe^3+^ ions by Mg^2+^ and Fe^2+^ ions in the octahedral sheet leading to the negative surface charge of MMT [28].

FTIR and XRD analyses were also applied for material characterization (the results are discussed in the adsorption mechanism part).

### 2.2. Results of the Adsorption Studies

#### 2.2.1. Effects of Adsorbent Dose

**Figure 1:**
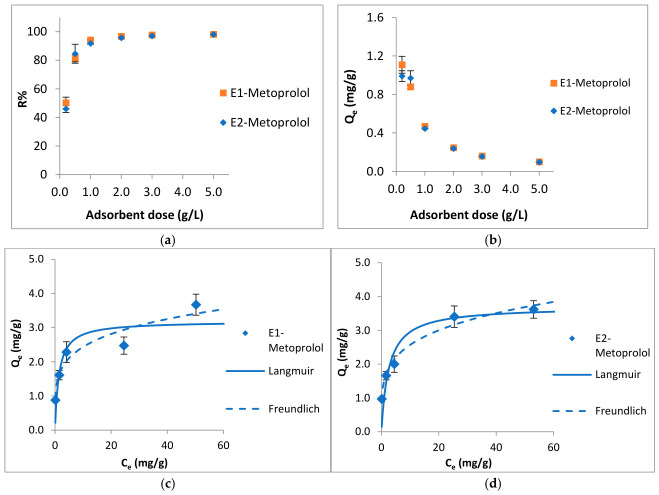
Adsorption of metoprolol by MMT: (**a**) effects of adsorbent dose on removal percent; (**b**) effects of adsorbent dose on Q_e_; (**c**) adsorption isotherm of E1-metoprolol; (**d**) adsorption isotherm of E2-metoprolol.

The effects of different adsorbent doses in the range of 0.2–5 g/L were evaluated to determine the optimum adsorption conditions. The results for metoprolol are given in Figure 1a,b. From this figure, it is seen that when the adsorbent dose increases, the amount of CPs adsorbed per unit weight of adsorbent at equilibrium (Q_e_) decreases. A similar trend can be observed for the other compounds (Figure S5). The decrease in the Q_e_ value with increase in the adsorbent dose can be attributed to the unsaturated adsorbent surface at high solid loading and the aggregation of the adsorbent. On the other hand, removal percent (R%) increases with increasing adsorbent dose, which can be expected by the more surface-active sites available at a high adsorbent dose. When the adsorbent dose was equal to or higher than 2 g/L, high removal efficiencies (88.0 to 99.8%) were obtained for the target CPs (Figure S5).

The results in Figure 1a,b and Figure S5 indicate that R% and Q_e_ obtained for each enantiomer pair are close to each other. In order to identify any significant difference, Student’s paired *t*-test was applied to the adsorption capacity and removal percentage data for the comparison of both enantiomers of the target compounds (Table S1). Based on the Student’s paired *t*-test results, the t values calculated for each target compound were lower than the critical *t* value of 3.106 (α = 0.01, df = 11). These results demonstrate that both enantiomers (E1 and E2) of the target compounds exhibit no significant difference in the adsorption capacities (Q_e_) and removal percentages. This lack of stereoselectivity is fundamentally tied to the adsorption mechanism elucidated in this study (Section 2.2.7). Raw Montmorillonite (MMT) K10 is an achiral mineral. Material characterization analyses (zeta potential, FTIR, and XRD) confirmed that the adsorption mechanism is based on electrostatic attraction, cation exchange, and hydrogen bonding interactions. Because these processes act uniformly on both mirror-image structures, they completely overshadow any subtle, potential chiral recognition forces. Though enantioselectivity is desirable for separation applications in analytical chemistry, an effective adsorbent for the removal of both enantiomers of chiral pollutants is preferable for water and wastewater treatment processes. Consequently, the non-stereoselective behavior of MMT, combined with its high adsorption potential, represents a major advantage regarding environmental remediation applications.

#### 2.2.2. Isotherm and Kinetic Studies

Equilibrium adsorption studies were conducted with different initial CP concentrations, and isotherm model fit was applied using Langmuir and Freundlich models. The results for metoprolol are given in Figure 1c,d and the results for the other compounds are shown in Figure S6. The maximum amounts adsorbed (experimental) were 3.7, 3.6, 9.3, 11.2, 6.1, 5.2, 42.1, 10.1, 9.0, 32.3, 33.1, 4.3, and 4.5 mg/g for E1-metoprolol, E2-metoprolol, E1-propranolol, E2-propranolol, E1-O-desmethylvenlafaxine, E2-O-desmethylvenlafaxine, norfluoxetine, E1-venlafaxine, E2-venlafaxine, E1-fluoxetine, E2-fluoxetine, E1-atenolol, and E2-atenolol, respectively.

Two common isotherm models were evaluated for the experimental data and the nonlinear model fit results are shown in Table S2. For the target compounds, the Freundlich model fitted well the experimental results compared to the Langmuir model, giving lower NRMSE and χ^2^ values, and higher R^2^. These results suggest that multilayers are formed on the MMT’s surface according to Freundlich model (rather than monolayer adsorption as described by the Langmuir model). The Freundlich parameter (n) was lower than the unity for each target CP (Table S2) indicating favorable adsorption [29]. The low n values (0.17–0.55) reported in Table S2 together with poor Langmuir model fits (R^2^ as low as 0.75) confirm strongly nonlinear, heterogeneous adsorption.

The effects of contact time were assessed by the adsorption experiments using different contact times (from 0.5 min to 24 h). The obtained results are given in Figure S7. The kinetic studies show that the adsorption equilibrium was attained at a contact time around 180 min (or lower). Three common kinetic models (pseudo-first order (PFO), pseudo-second order (PSO), and Elovich) were used to correlate the kinetic data. The model parameters with kinetic data are depicted in Table S3. For most of the compounds, the Elovich model provided a better fit when compared to the other models. For E1-O-desmethylvenlafaxine, E2-O-desmethylvenlafaxine, and norfluoxetine, both PSO and Elovich models showed good agreement with the experimental data. It is worth noting that some studies in the literature relate PSO and Elovich models to the chemical adsorption process; however, this approach was criticized since the kinetic model fit is not enough to explain adsorption mechanisms, and more sophisticated methods (based on material characterization) are needed to understand the adsorption mechanisms [29]. For the kinetic results in this study, the Elovich model fits well, which is consistent with (but does not prove) the electrostatic attraction mechanism supported by FTIR and zeta potential analyses (discussed in Section 2.2.7).

#### 2.2.3. Effects of Solution pH

**Figure 2:**
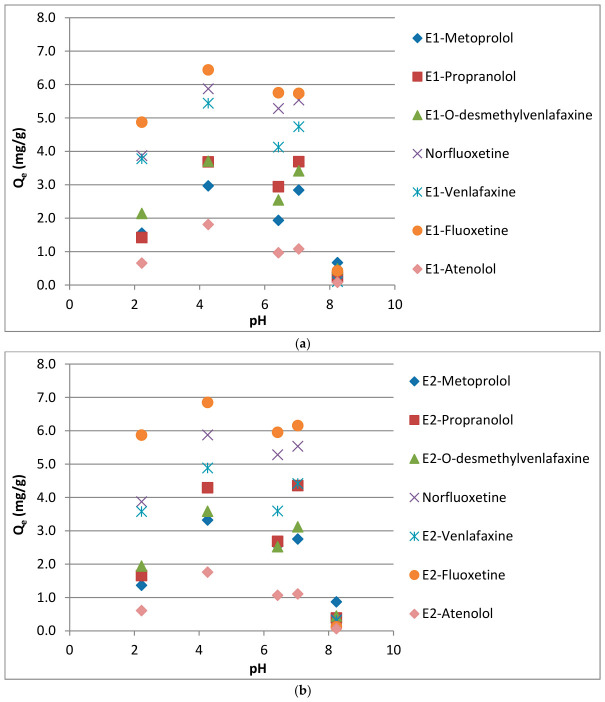
Effects of solution pH on the amounts adsorbed for the target CPs (**a**) E1-enantiomers; (**b**) E2-enantiomers (error bars are not shown for clarity; RSD < 5%).

The effects of solution pH on the equilibrium adsorption amount per unit weight of adsorbent (Q_e_) were studied by performing adsorption experiments at different equilibrium solution pH (between 2 and 8). The results for E1 and E2 enantiomers of the target CPs are shown in Figure 2a and Figure 2b, respectively. For each compound, Q_e_ values are high and almost constant in the acidic and neutral pH range, but they decrease sharply at the basic pH range. This observation can be attributed to the surface charge of MMT and pH-dependent speciation of the target compounds.

As shown in Figure S4, the zeta potential is negative for MMT under the studied pH range, suggesting that the surface of MMT is negatively charged. This negative charge is explained by the isomorphic substitution (i.e., Al^3+^ are substituted by Mg^2+^ in the octahedron and Si4+ are substituted by Al^3+^ in the tetrahedron), leading to a permanent negative charge on the MMT layers [30]. On the other hand, the pK_a_ values of the target CPs are between 8.9 and 9.8 [31,32] as shown in Table S4. As a result, these compounds are predominantly positively charged under acidic and neutral pH conditions, leading to their attraction by the negatively charged MMT surface. Figure S4 also demonstrates that the MMT surface slightly becomes less negative after the adsorption process (especially between pH 6 and 9). This result is due to the adsorption of positively charged CP molecules by the negatively charged MMT surface, leading to the slightly less negative surface charge of MMT. Thus, it can be concluded that the adsorption of positively charged molecules balanced some of the negative surface charge on the clay mineral [33].

Figure 2 also shows that as the solution pH becomes basic, Q_e_ values for the target CPs decrease. Since the neutral species of the target CPs become predominant at high pH values, the interaction between the permanently negatively charged MMT surface and the target CP molecules is reduced, leading to a decrease in the adsorbed amounts under alkaline conditions.

#### 2.2.4. Studies Using Real Water and Wastewater

To determine adsorption efficiency using real water and wastewater, adsorption experiments with tap water, surface water, and wastewater were conducted. Tap water was obtained within Seville City, the surface water was taken from the Guadalquivir River, and the wastewater effluent was from a wastewater treatment plant in Seville (Spain). A 0.5% (*v*/*v*) acetonitrile solution was added for sample stabilization, and the samples were stored in a refrigerator until analysis. To remove suspended solids, filtration was applied by 1.2 μm glass fiber filters (Whatman, Maidstone, UK).

The results of the CP adsorption experiments with real water and wastewater are shown in Figure S8. In this figure, it can be deduced that the amounts adsorbed at equilibrium (Q_e_) are almost constant (or slightly decreased) in tap water for the target CPs. Interestingly, when surface water is used in the adsorption experiments, there are some increases in Q_e_ values for each target CP, except for fluoxetine and norfluoxetine. The observed increase in Q_e_ values (up to 10.5%) can be explained by humic acid (HA) in surface water, which can participate in complex formation process with the target CPs. To further investigate the effects of solution HA content, adsorption experiments with HA-containing solutions were also applied, which will be discussed in the next section.

When real wastewater was used in the adsorption experiments, decreases in Q_e_ values (between 20.6 and 34.1%) were observed for the target CPs (Figure S8). This decrease can be attributed to the wastewater’s complex organic and inorganic constituents, which compete with the CP molecules for the active adsorption sites and partially block the pores on the MMT surface. Similar decreases in the adsorption efficiencies were reported for different adsorbent materials used in pharmaceutical removal from real wastewater [34,35].

#### 2.2.5. Effects of Humic Acid Content

**Figure 3:**
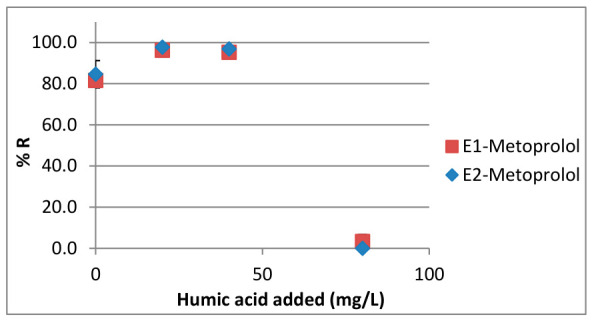
Effects of solution HA content on the adsorption of metoprolol.

The effects of solution humic acid (HA) content on the adsorption of target CPs are given in Figure S9. Increasing the solution HA content up to 40 mg/L led to increases in the removal efficiencies for most of the target compounds (except fluoxetine and norfluoxetine). However, further increases in the solution HA content resulted in lower removal efficiencies for these compounds. For example, as depicted in Figure 3, the percent removal was 81.6% for E1-metoprolol when no HA was added to the solution. The removal efficiency increased to 96.3% and 95.3% when the solution HA concentration was increased to 20 and 40 mg/L, respectively. Further increase in the HA concentration to 80 mg/L resulted in removal efficiency close to zero. A similar trend was observed for other target CPs (except fluoxetine and norfluoxetine).

The complex formation mechanism can explain the increased adsorption amounts obtained by HA addition to the solution. Zhao et al. (2012) observed a similar increase in the adsorbed amounts of cationic and zwitterionic forms of tetracycline by MMT in the presence of HA [36]. This result was attributed to the complexation of cationic and zwitterionic species of tetracycline with the deprotonated sites on HA via electrostatic attraction. In the current study, the target CPs are also positively charged under neutral pH conditions (Table S4). HA with electron-withdrawing aromatic moieties can form complex with positively charged CPs, which enhance the removal of these compounds. Thus, the overall removal of CPs includes their direct adsorption on the MMT surface and complex formation with HA.

On the other hand, further increasing the HA concentration leads to reduced adsorption amounts. Lian et al. (2015) also observed a similar trend where sulfanilamide adsorption on biochar slightly increased before decreasing with the presence of HA [37]. This behavior was explained by the structural variations in the adsorbed HA. During the sorption process of HA, the innermost molecular layers of HA (close to the adsorbent surface) form a highly condensed structure due to the attractive force of the adsorbent surface. This condensed zone provides some additional sorption sites or a partitioning phase for the target pollutants that mildly enhances their overall adsorption [38]. On the other hand, as HA loading increases, weaker surface attractions at greater distances (away from the adsorbent surface) cause the outer HA layers to expand and this significantly reduces the adsorption of the target molecules at elevated HA concentrations [37]. These findings indicate that HA exerts conflicting (facilitating and inhibiting) effects on the adsorption process which is governed by HA concentration in the aqueous medium.

The cases for fluoxetine and norfluoxetine were different, where an increase in the solution HA content constantly lowered Q_e_ values (Figure S9). Without HA addition, fluoxetine and norfluoxetine already showed very high adsorption capacities (Table S2), and the addition of HA caused a decrease in Q_e_ values for these compounds. In general, two-sided effects of HA can be expected on adsorption behavior: (1) enhanced adsorption due to the formation of additional partition sites in the presence of HA; and (2) lower adsorption capacities resulting from pore blockage and competitive adsorption between HA and the target pollutants on the solid surface. Because fluoxetine and norfluoxetine possess larger molecular sizes (compared to the other target pollutants in this study), their adsorption can be more severely affected by the pore blockage and competitive adsorption mechanisms of HA. As indicated by Jin et al. (2018), the competitive effects of HA for the adsorption sites can exert greater impact for larger molecular weight compounds [39]. Zhang et. al. (2018) also observed a decrease in the adsorption capacity of norfluoxetine on a montmorillonite modified biochar composite in the presence of HA [40]. This observation is explained by HA blocking the adsorption sites on the adsorbent material and competing with norfluoxetine for these active sites.

#### 2.2.6. Desorption and Reuse

The reusability is essential to assess the suitability of an adsorbent for sustainable application. Regarding this, four adsorption-desorption cycles were tested for MMT, and the amounts of the target CPs adsorbed per unit weight of the adsorbent were calculated. The results are given in Figure S10. From these results, it can be inferred that for most of the target CPs, the reusability of MMT was very high, i.e., the percent adsorption capacities retained after four reuse cycles were 79.5, 81.8, 78.6, 77.3, 84.4, 80.6, 83.5, 63.8, 59.9, 77.8, 78.2, 81.4, and 87.5% for E1-metoprolol, E2-metoprolol, E1-propranolol, E2-propranolol, E1-O-desmethylvenlafaxine, E2-O-desmethylvenlafaxine, norfluoxetine, E1-atenolol, E2-atenolol, E1-fluoxetine, E2-fluoxetine, E1-venlafaxine, and E2-venlafaxine, respectively. The possible reasons for good reusability obtained for MMT will be discussed in the next section, along with the adsorption mechanism.

#### 2.2.7. Adsorption Mechanism

Surface properties of MMT showed several changes after the adsorption of the target CPs. Based on the scanning electron microscopy (SEM) results, MMT’s surface becomes less porous after adsorption, and dense aggregates can be observed (Figure S1). The BET SSA calculated from Figure S2 was reduced from 221 m^2^/g to 171 m^2^/g after the adsorption process. The average pore size of MMT was initially 6.76 nm and reduced to 3.5 nm after adsorption. The observed changes demonstrate that the target CPs were successfully adsorbed on the MMT surface.

**Figure 4:**
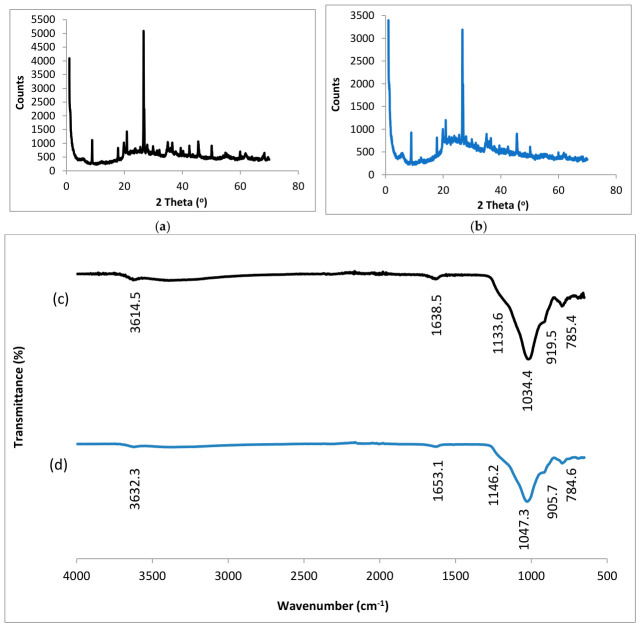
XRD and FTIR spectra of MMT: (**a**) XRD before adsorption; (**b**) XRD after adsorption; (**c**) FTIR before adsorption; (**d**) FTIR after adsorption.

XRD patterns of MMT before and after adsorption are described in Figure 4a and Figure 4b, respectively. Characteristic diffraction peaks of MMT were observed at 2θ = 5.81°, 19.86°, 35.04°, and 61.86° angles. These observations are consistent with other studies on MMT [41,42,43]. The peaks at 20.88°, 26.62°, and 36.57° were assigned to quartz. For MMT after adsorption, slight changes were observed in the XRD patterns. Decreases in the peak intensities of MMT were observed at 35.04° and 61.86°, which suggests that there is an interaction between the adsorbed molecules and the MMT surface. Moreover, higher noise observed in the XRD patterns for MMT after adsorption can be attributed to higher amorphous content [44] due to adsorbed CP molecules. On the other hand, there was no significant shift in the peak positions at 2θ values. This result indicates that the interlayer spacing of MMT was not altered after adsorption. As a result, the adsorption takes place on the outer surfaces and edges with no detectable intercalation.

FTIR spectra of MMT before and after adsorption of the target CPs are given in Figure 4c and Figure 4d, respectively. In Figure 4c, the characteristic bands for MMT can be observed, which are compatible with observations in the previous studies [45,46,47]. The band at 3614.5 cm^−1^ corresponds to the stretching vibrations of the structural hydroxyl groups (Al–OH) in the octahedral sheet. The O–H stretching and bending vibrations from adsorbed water are located at 3438.4 cm^−1^ and 1638.5 cm^−1^, respectively. The shoulder at 1133.6 cm^−1^ is assigned to out-of-plane Si–O stretching. The most intense band at 1034.4 cm^−1^ is due to Si–O in-plane stretching. The bending vibration of the Al_2_OH group in the octahedral layer of MMT is observed at 919.5 cm^−1^. The peak at 785.4 cm^−1^ is explained by the Si–O stretching of quartz, and the peak at 697.4 cm^−1^ is related to Si–O bending of quartz.

For MMT after the adsorption of CP molecules, the changes in the FTIR spectra can be observed in Figure 4d. Al–OH stretching vibrations at 3614.5 cm^−1^ became less intense and moved to 3632.3 cm^−1^ after adsorption. Moreover, the peak at 1034.4 cm^−1^ corresponding to the Si–O stretching (in-plane) was shifted to 1047.3 cm^−1^. Similarly, the peak at 1133.6 cm^−1^ for Si–O stretching (out-of-plane) was shifted to 1146.2 cm^−1^. These changes can be attributed to the interaction between negatively charged Si–O groups in the MMT structure and positively charged molecules of mixed enantiomers during adsorption. The peak at 919.5 cm^−1^ related to Al_2_OH bending was shifted to 905.7 cm^−1^, indicating that O–H groups in MMT structure were involved in the adsorption mechanism.

The results of material characterization analyses are integrated below to elucidate the adsorption mechanism. XRD data indicate that MMT’s interlayer spacing remained unchanged after adsorption. Consequently, the process takes place on external surfaces and edges without any detectable intercalation. BET analysis shows a decrease in both the SSA and the average pore size of MMT after adsorption, which supports the occurrence of surface adsorption process.

Zeta potential analyses demonstrate that MMT’s surface is negatively charged under the studied pH range (2–11). On the other hand, the target CPs predominantly exist in protonated form below their pKa values, which are between 8.9 and 9.8 (Table S4). Thus, electrostatic attraction is expected between the MMT surface and the positively charged CPs. Moreover, cation exchange interaction is also among the proposed mechanisms for positively charged molecules by MMT [36,48]. As explained in the previous part, the zeta potential of MMT becomes less negative after the adsorption process, which supports both electrostatic attraction and cation exchange interaction [49]. Notably, the amounts adsorbed are almost zero at high pH levels (where the target CPs exist in their neutral form). Therefore, hydrophobic interactions can be excluded from the proposed adsorption mechanism.

FTIR spectra recorded before and after adsorption reveal that Si–O and Al–OH groups of MMT participate in the adsorption mechanism. Consequently, the plausible mechanism involves hydrogen bonding between the functional groups of the target CPs (i.e., amine, fluorine, hydroxyl groups) and the Si–O and Al–OH groups of MMT.

In summary, the observed results from material characterization indicate that the adsorption mechanism is based on electrostatic attraction, cation exchange, and hydrogen bonding interactions. On the other hand, the observed changes were not intense, corresponding to weak interactions and surface adsorption. These properties are preferable when the adsorbent’s reusability is considered, and the high reuse potential of MMT for the target CPs can be attributed to this weak surface adsorption.

#### 2.2.8. Limitations and Future Perspectives

Although MMT shows high removal efficiencies for both enantiomers of CPs, its reduced adsorption capacity in real wastewater may restrict its practical applications. Additionally, despite the promising results of the reuse studies, solvent consumption and the generation of secondary waste containing the target pollutants are among the main environmental considerations. Based on these aspects, evaluating the scale-up feasibility and conducting complete life cycle assessment can be considered as future research directions.

## 3. Materials and Methods

### 3.1. Materials

High purity standards of atenolol, fluoxetine, metoprolol, norfluoxetine, O-desmethylvenlafaxine, propranolol, and venlafaxine were purchased from Sigma-Aldrich (Steinheim, Germany). The physicochemical properties of these compounds are shown in Table S4. The standards were dissolved in methanol to make 1000 ppm standard solutions. Using these solutions, aqueous phase mixtures of the CPs were prepared at different concentrations. Montmorillonite K10, humic acid, and ammonium formate were supplied by Sigma-Aldrich (Steinheim, Germany). All the solvents used in the analyses were of HPLC grade (or better) and purchased from Romil Ltd. (Barcelona, Spain). Other chemicals and reagents were of analytical quality and were obtained from Panreac (Barcelona, Spain).

### 3.2. Methods

#### 3.2.1. Adsorbent Characterization

Characterization of MMT was performed by scanning electron microscopy (SEM), Fourier-transformation infrared spectroscopy (FTIR), X-ray diffraction (XRD), zeta potential, thermogravimetric analysis (TGA), and nitrogen adsorption and desorption experiments.

SEM analyses were carried out by a FEI-TENEO electron microscope (FEI Co., Hillsboro, OR, USA). For FTIR analyses, a Tensor II spectrometer (Bruker, Karlsruhe, Germany) was used in the spectral region of 4000–650 cm^−1^ and 4 cm^−1^ spectral resolution. XRD tests were conducted by Bruker D8 Advance A25 diffractometer (Bruker Inc., Karlsruhe, Germany) at 40 kV and 30 mA, with a Cu-Kα radiation value of 1.5418 Å. The diffraction angle (2θ) range was between 1° and 70° with step size and time set to 0.03° and 0.1 s, respectively. The zeta potential of samples was tested by a Zetasizer Nanosystem (Malvern Inst., Southborough, MA, USA) at 25 °C. Prior to zeta potential analyses, MMT was dispersed in water (0.1 g/L), and pH was adjusted to different values (ca. 2, 4, 6, 9, 11) by using 0.1 M NaOH or HCl. Dispersions were mixed during 24 h for pH stabilization, and then, the zeta potential was measured. Thermogravimetric analysis was applied by a Q600 STD (TA Inst., New Castle, DE, USA) with 10 °C/min heating from 20 °C to 600 °C under a N_2_ atmosphere. Specific surface area (SSA) of MMT was assessed by the N_2_ adsorption/desorption technique at a low temperature (77 K) using a gas sorption analyzer (ASAP 2420, Micromeritics, Norcross, GA, USA).

#### 3.2.2. Adsorption Studies

Adsorption experiments were applied in batch mode by modifying one variable at a time. The details of the adsorption conditions throughout the studies are given in Table S5, and they are summarized below. In a typical adsorption experiment, 10 mL aqueous phase mixed CP solutions were prepared with a concentration of 1 mg/L, corresponding to 0.5 mg/L of each enantiomer. (The CP concentrations were selected to have environmentally relevant, i.e., low initial concentrations and to have final concentrations within the calibration limits after adsorption). MMT was added at a dose of 0.5 g/L (chosen based on the results of dose experiments). The samples were stirred for 24 h under ambient temperature (25 + 1 °C) using a multi-stirrer magnetic device at 350 rpm (Selecta, Multimatic 9N, Abrera, Spain). Samples without the addition of MMT (control) were also prepared.

The typical conditions described above were used in the adsorption experiments unless otherwise stated. As indicated in Table S5, the effect of the adsorbent dose was assessed by applying MMT doses in the range of 0.2–5 g/L (keeping the other parameters constant). For the isotherm studies, initial adsorbate concentrations in the range of 1–100 mg/L were applied (corresponding to a range of 0.5–50 mg/L for each enantiomer). Although this range is higher than environmentally relevant concentrations, in this study, the lower bound (0.5 mg/L) was chosen to be able to measure the remaining concentration after the adsorption process. A high upper limit (50 mg/L) was chosen to have a wide range of concentrations and to obtain a complete adsorption isotherm, which is described as the ideal approach for determining isotherm parameters [50]. As indicated in a previous study, plotting Q_e_ versus C_e_ for the complete adsorption isotherm is essential for identifying the regions where the experimental data relating to adsorption equilibrium are actually located [29].

The effects of pH were examined by performing the adsorption experiments using different equilibrium solution pH values (2 to 8). The initial pH values were adjusted using 0.1 M NaOH or HCl. The reported pH values indicate the equilibrium values. (Higher pH values were not applied since the amounts adsorbed for the target CPs were almost zero at basic solution pH, which is discussed in Section 2.2.3). The effects of humic acid (HA) were determined by preparation of the solutions containing 0, 20, 40, and 80 mg/L HA and then conducting the adsorption experiments with these solutions. Adsorption experiments with tap water, surface water, and wastewater were performed by adding 5 mg MMT into 10 mL samples containing 1 mg/L mixed enantiomers (i.e., 0.5 mg/L of each enantiomer). For desorption and reuse studies, the adsorbent is collected after the adsorption experiment, washed with 10 mL methanol, centrifuged, and rinsed with deionized water. The collected MMT was dried at 100 °C oven and desiccated prior to reuse. This desorption–reuse cycle was applied four times to assess the reusability of the adsorbent.

For the kinetic experiments, different contact times were applied between 0.5 min to 24 h using a solution volume of 200 mL and an initial adsorbate concentration of 10 mg/L (i.e., 5 mg/L of each enantiomer). At the end of specified time intervals, 1 mL aliquots were taken from the solution and prepared for liquid chromatography-tandem mass spectrometry (LC-MS/MS) analysis, as described in the next section.

#### 3.2.3. Analytical Methods

Samples were filtered using syringe filters (0.22 µm) after adsorption and analyzed by LC-MS/MS. A highly sensitive and validated analytical method was utilized for this purpose [51]. Enantiomeric separation and quantification were achieved using an Agilent 1260 Infinity II chromatograph coupled to a 6495 triple quadrupole mass spectrometer (LC-MS/MS) equipped with a specialized chiral stationary phase column (Supelco Astec Chirobiotic V, Sigma-Aldrich, St. Louis, MO, USA). The method operated under a rigorous 6-point calibration system. The volume of injection was 5 μL and the column temperature was 30 °C. The mobile phase was 10 mM ammonium acetate buffer (pH adjusted to 4 by formic acid) in water (solvent A) and methanol (solvent B) with a flow rate of 0.4 mL/min. Acquisition was set in a multiple reaction monitoring (MRM) mode. The details of method parameters and coefficient of determination (R^2^) obtained for the calibration curves are given in Table S6.

#### 3.2.4. Results Evaluation

After the analytical separation, the first and last eluting enantiomers were denoted as E1 and E2. Chiral separation of norfluoxetine could not be achieved, and the total concentrations of both enantiomers were reported for this compound. The measured concentrations were used to calculate the amounts adsorbed per unit weight of the adsorbent at time t (Q_t_) and percent removal efficiency (R%), which are given in Equations (1) and (2), respectively.

(1) $$Q_{t}=\frac{\left(C_{0}-C_{t}\right)V}{m}$$

(2) $$R\%=\frac{C_{0}-C_{t}}{C_{0}}\times 100\%$$

In these equations, C_0_ is the initial CP concentration (mg/L); C_t_ is the CP concentration at any time (mg/L); V is the solution volume (L); and m is the weight of the adsorbent (g).

The adsorption experiments were performed as independent duplicates. The reported values are the averages of duplicates and that the relative standard deviation (RSD) between replicates was lower than 5%. Isotherm and kinetic models were fitted to the experimental data by nonlinear optimization technique using Solver function in Excel spreadsheet (Microsoft Corp., Redmond, WA, USA) [29]. Goodness-of-fit for the models was determined by computing the normalized root mean square error (NRMSE), coefficient of determination (R^2^), and chi-square (χ^2^), which are presented in Table S7.

## 4. Conclusions

In this study, montmorillonite (MMT) was evaluated for the simultaneous adsorption of chiral pharmaceutical (CP) mixture in aqueous solutions, including atenolol, fluoxetine, metoprolol, norfluoxetine, O-desmethylvenlafaxine, propranolol, and venlafaxine. The obtained results are summarized below:

- Adsorption studies demonstrate that MMT can be used as a low-cost, non-enantioselective adsorbent, which is effective for the simultaneous removal of both enantiomeric forms of several chiral pharmaceuticals, thereby simplifying treatment monitoring and validation.
- The sorption isotherms followed the Freundlich model rather than the Langmuir model, and the process was strongly nonlinear, heterogeneous, and favorable. The Elovich kinetic model adequately represented the experimental results for most of the target compounds.
- At acidic and neutral solution pH levels, the adsorption efficiency of MMT was high for each target CP and the amounts adsorbed decreased drastically at basic pH conditions. These observations were explained by the pH-dependent speciation of the target compounds and the negative surface charge of MMT under the studied pH conditions.
- When real water samples were used, the adsorption amounts showed almost no change (or slight decrease) in tap water, while enhanced adsorption was observed in the surface water for most of the target CPs. When wastewater was used, the amounts adsorbed decreased for each compound (up to 34.1%).
- For most of the target CPs, an increase in the solution HA content up to 40 mg/L led to an increase in the percent removal efficiencies but further increase in HA content caused decreases in the removal efficiencies.
- Four cycles of desorption and reuse studies indicated that for most of the target CPs, the reusability of MMT was high, with only 12.5 to 22.7% reduction in the initial adsorption amounts.
- The suggested adsorption mechanism is based on electrostatic attraction, cation exchange, and hydrogen bonding interactions.

## Data Availability

Data will be made available on request.

## Supplementary Materials

The following supporting information can be downloaded at: https://www.mdpi.com/article/10.3390/molecules31122040/s1, Figure S1: SEM images of MMT. (a) before adsorption (b) after adsorption; Figure S2: BET adsorption-desorption isotherms. (a) MMT before adsorption (b) MMT after adsorption; Figure S3: TGA thermogram of MMT; Figure S4: pH versus zeta potential of MMT before and after adsorption; Figure S5: Effects of adsorbent dose (a) %R for metoprolol (b) Q_e_ for metoprolol (c) %R for propranolol (d) Q_e_ for propranolol (e) %R for O-desmethylvenlafaxine (f) Q_e_ for O-desmethylvenlafaxine (g) %R for norfluoxetine (h) Q_e_ for norfluoxetine (i) %R for venlafaxine (j) Q_e_ for venlafaxine (k) %R for fluoxetine (l) Q_e_ for fluoxetine (m) %R for atenolol (n) Q_e_ for atenolol; Table S1: Paired sample *t*-test results for percent removal and Q_e_ for E1 and E2 (α= 0.01, df= 11, t_Critical= 3.106); Figure S6: Equilibrium data and isotherm model fit (a) E1-Metoprolol (b) E2-Metoprolol (c) E1-Propranolol (d) E2-Propranolol (e) E1-O-desmethylvenlafaxine (f) E2-O-desmethylvenlafaxine (g) Norfluoxetine (h) E1-Venlafaxine (i) E2-Venlafaxine (j) E1-Fluoxetine (k) E2-Fluoxetine (l) E1-Atenolol (m) E2-Atenolol; Table S2: Isotherm model fit parameters; Figure S7: Kinetic results and model fit for E1-Metoprolol, E2-Metoprolol, E1-Propranolol, E2, Propranolol, E1-Venlafaxine, E2-Venlafaxine, E1-O-desmethylvenlafaxine, E2-O-desmethylvenlafaxine, Norfluoxetine (error bars are not shown for clarity; RSD < 5%); Table S3: Kinetic model fit parameters; Table S4: Physical and chemical properties of the target pharmaceuticals used; Figure S8: Effects of deionized water (DIW), tap water, surface water and wastewater (a) Propranolol (b) Metoprolol (c) Fluoxetine (d) O-desmethylvenlafaxine (e) Venlafaxine (f) Norfluoxetine (g) Atenolol; Figure S9: Effects of humic acid on the adsorption of (a) Propranolol (b) Metoprolol (c) Fluoxetine (d) O-desmethylvenlafaxine (e) Venlafaxine (f) Norfluoxetine (g) Atenolol; Figure S10: Four-cycle desorption and reuse results (error bars are not shown for clarity; RSD < 5%); Table S5: Experimental conditions used in adsorption studies*; Table S6: LC–MS/MS method parameters and coefficient of determination (R2) of calibration; Table S7: Parameters used for model evaluation.
