# Supplementary material for: Adsorption Behavior of Chiral Pharmaceuticals onto Montmorillonite Clay: Evaluating Removal Efficiency and Stereoselectivity
Source: Molecules. 2026 Jun 11;31(12):2040. doi: 10.3390/molecules31122040 (PMC13304480; doi:10.3390/molecules31122040)
Supplement: Supplementary file 1 [file molecules-31-02040-s001.zip › molecules-4333294-supplementary.pdf]

Supplementary materials

Adsorption Behavior of Chiral Pharmaceuticals onto Montmorillonite Clay: Evaluating Removal Efficiency and Stereoselectivity

Gül Gülenay Hacıosmanoğlu [1,*], Marina Arenas [2], Carmen Mejías [2], Julia Martín [2,*], Juan Luis Santos [2], Irene Aparicio [2] and Esteban Alonso [2]

[1] Environmental Engineering Department, Faculty of Engineering, Marmara University, Uyanık Cd. No:6, 34840 Istanbul, Turkey
[2] Departamento de Química Analítica, Escuela Politécnica Superior, Universidad de Sevilla, C/Virgen de África 7, E-41011 Sevilla, Spain
* Correspondence: gulenay.haciosmanoglu@marmara.edu.tr (G.G.H.); jbueno@us.es (J.M.)

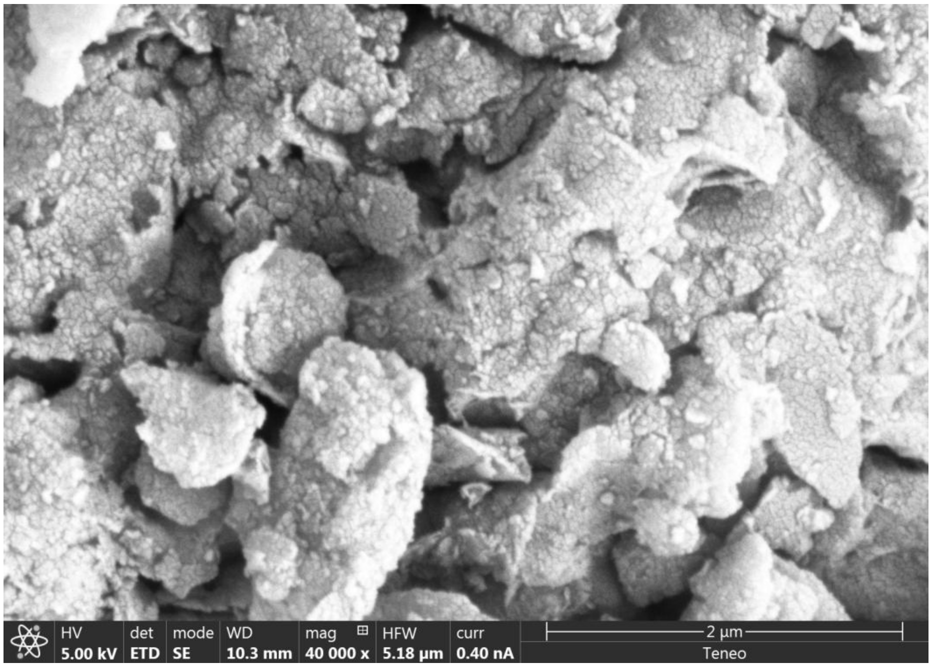

(a)

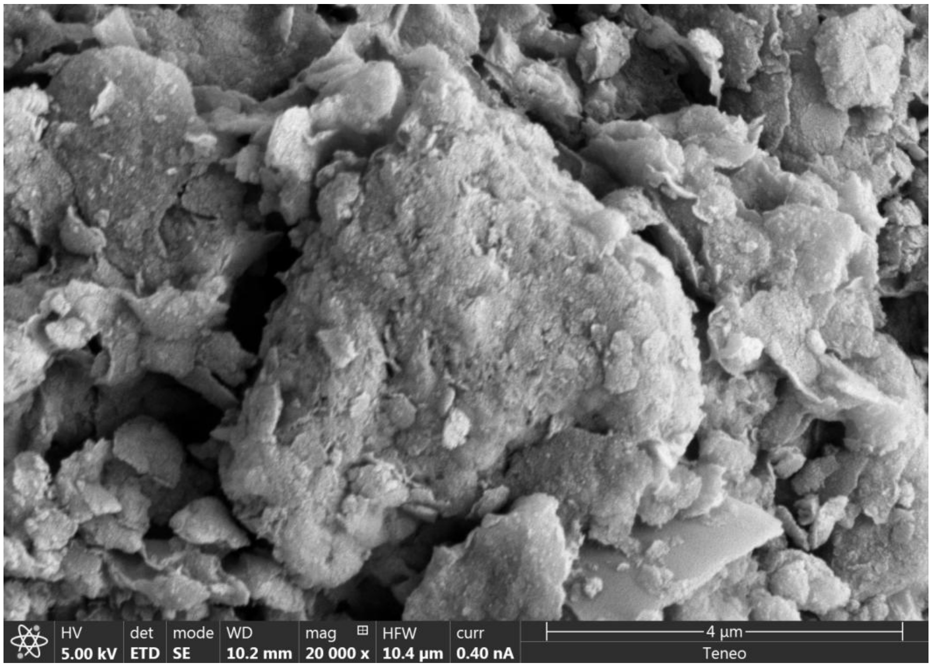

(b)

Figure S1. SEM images of MMT
(a) before adsorption (b) after adsorption

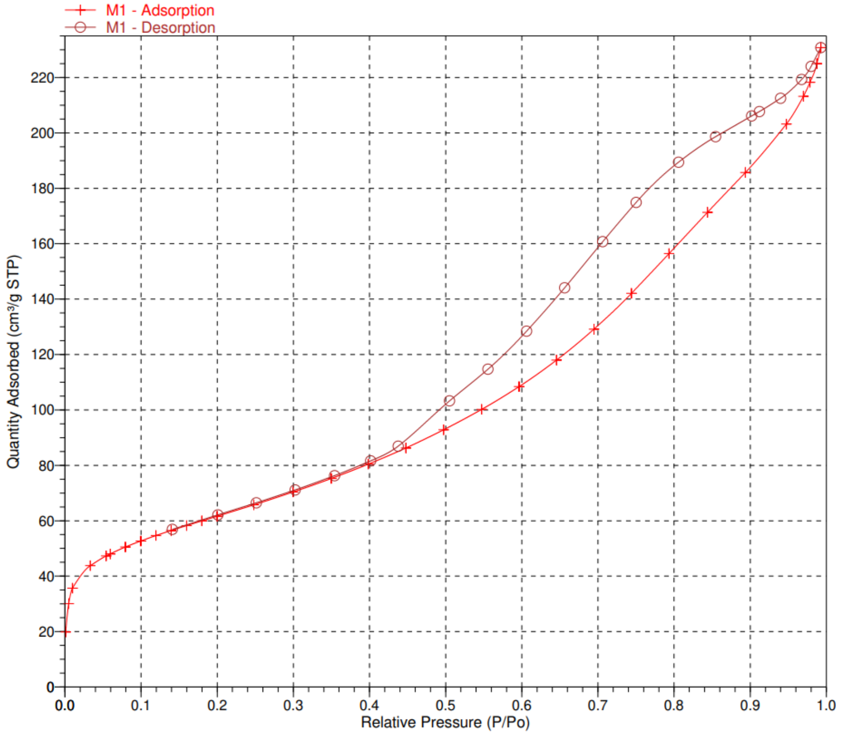

(a)

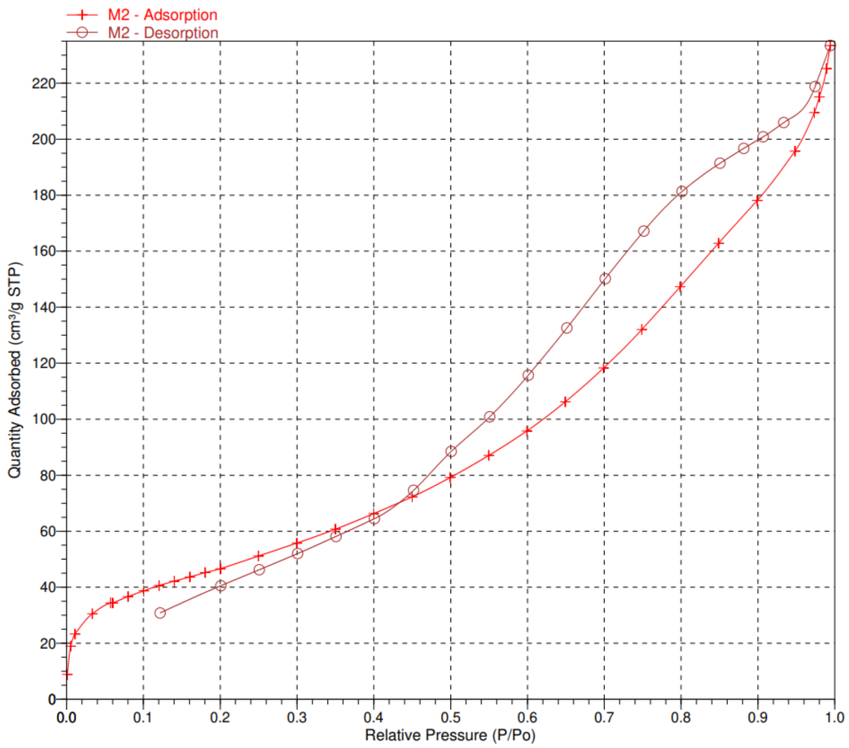

(b)

Figure S2. BET adsorption-desorption isotherms

(a) MMT before adsorption (b) MMT after adsorption

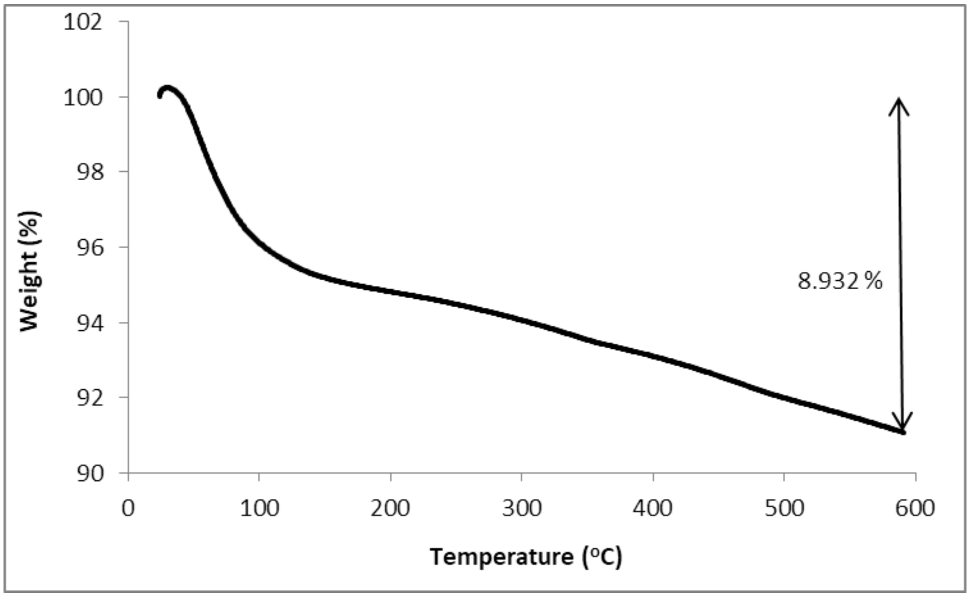

Figure S3. TGA thermogram of MMT

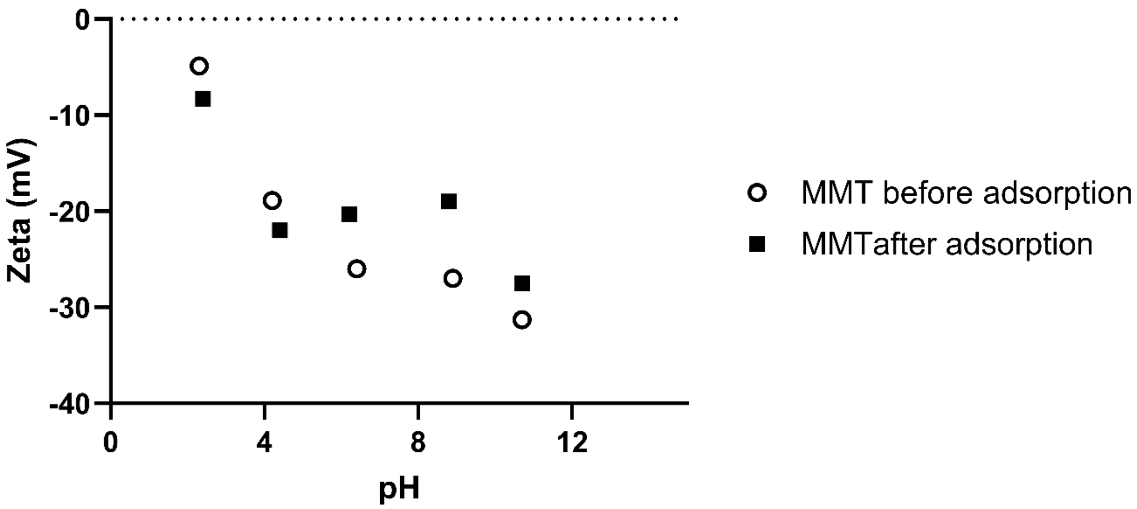

Figure S4. pH versus zeta potential of MMT before and after adsorption

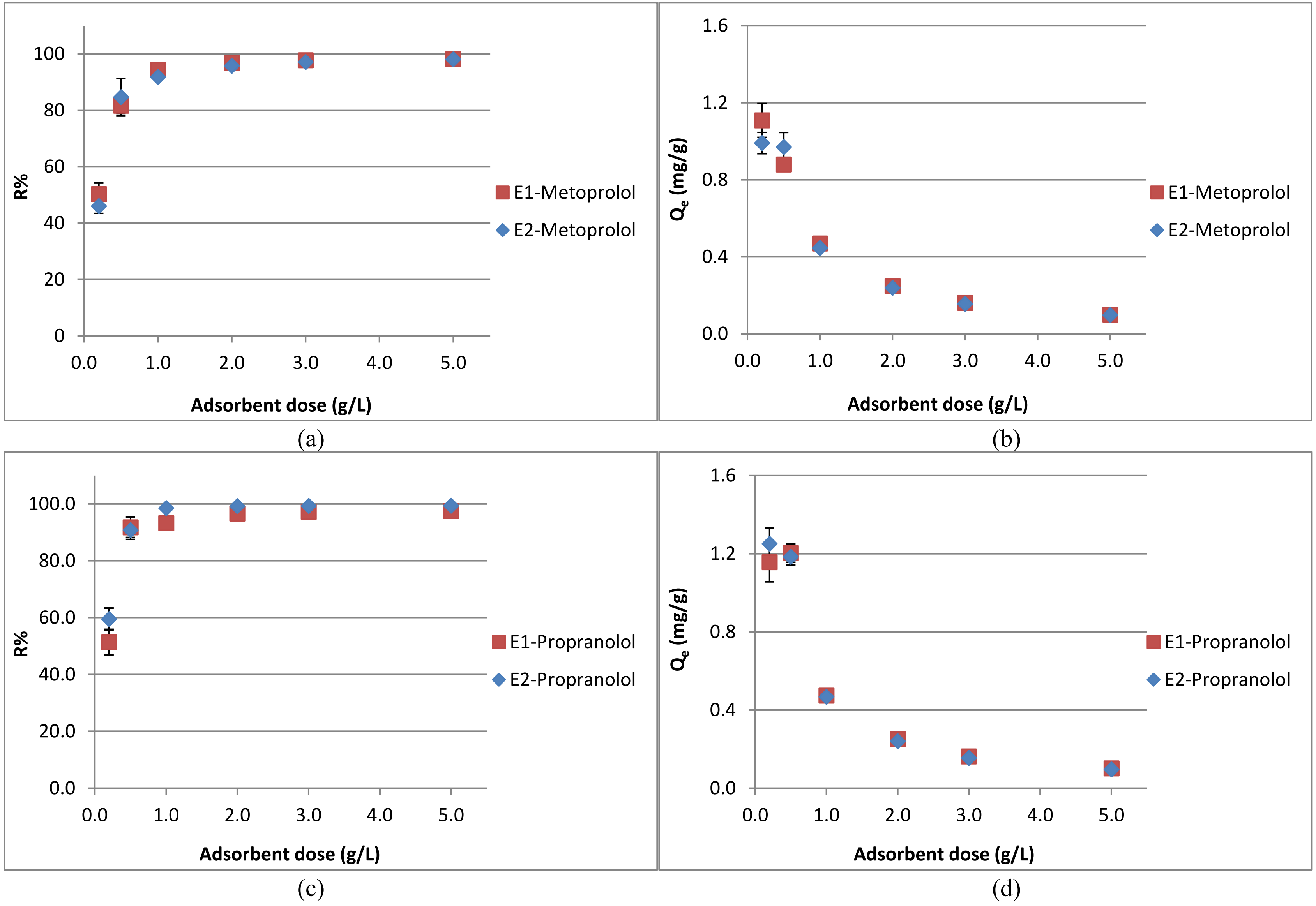

Figure S5. Effects of adsorbent dose (a) %R for metoprolol (b) Q_e for metoprolol (c) %R for propranolol (d) Q_e for propranolol

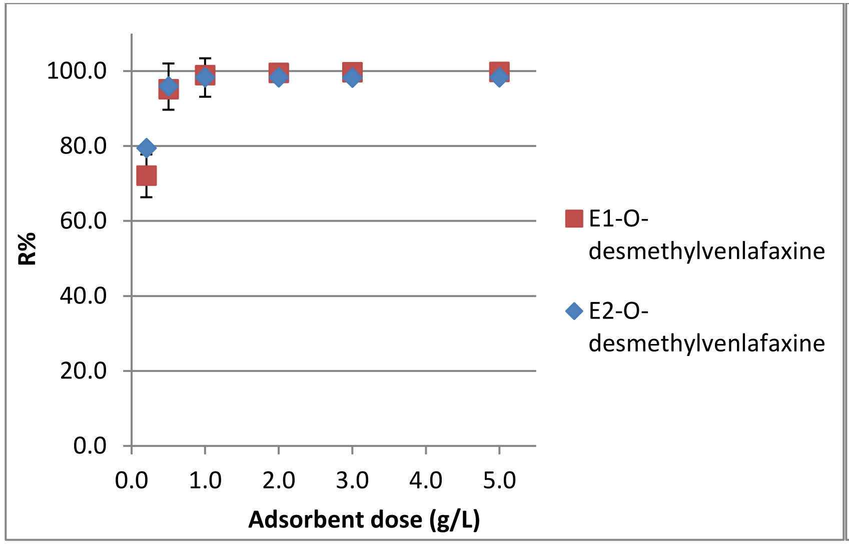

(e)

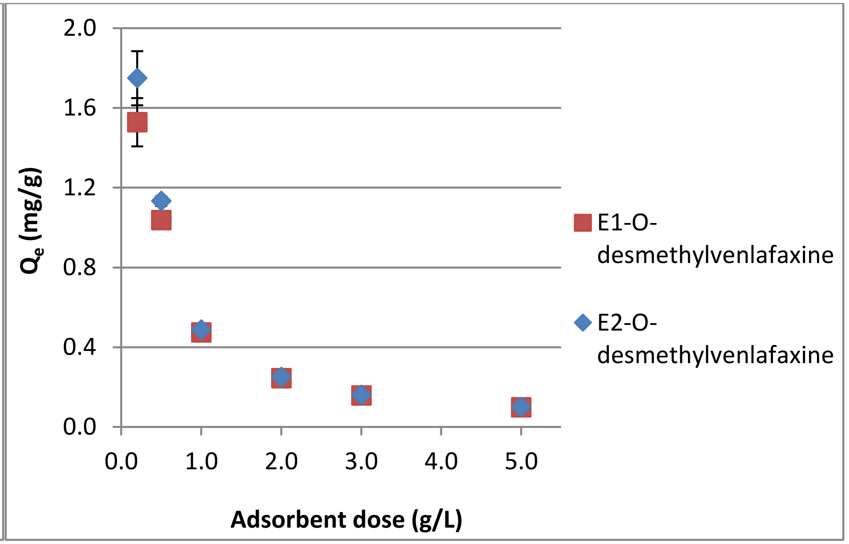

(f)

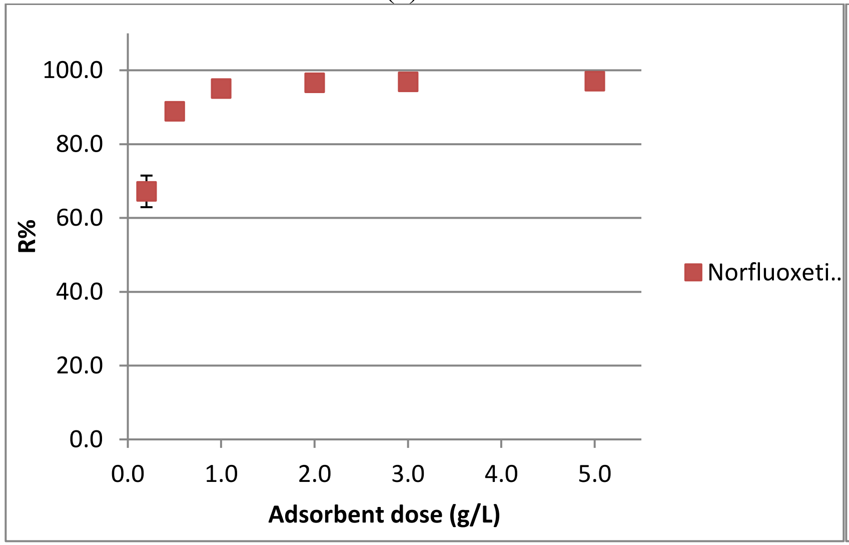

(g)

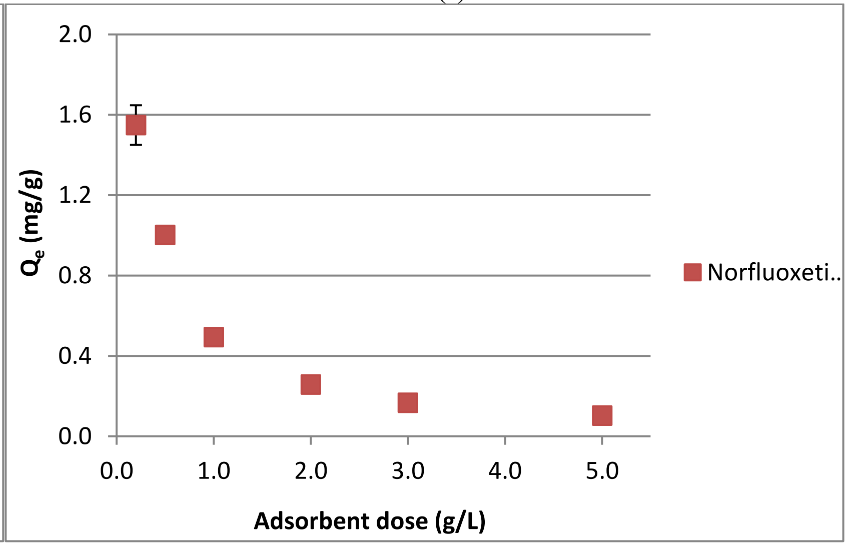

(h)

Figure S5 (continued). Effects of adsorbent dose (e) %R for O-desmethylvenlafaxine (f) Q_e for O-desmethylvenlafaxine (g) %R for norfluoxetine (h) Q_e for norfluoxetine

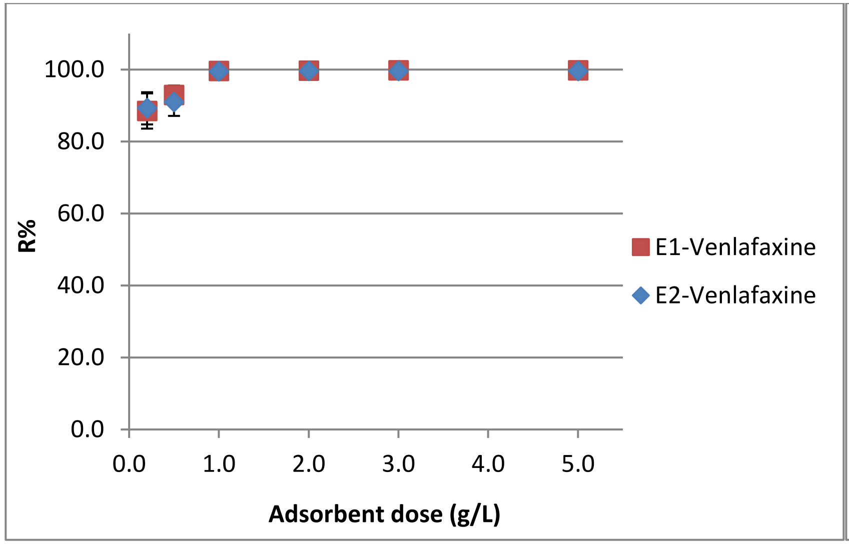

(i)

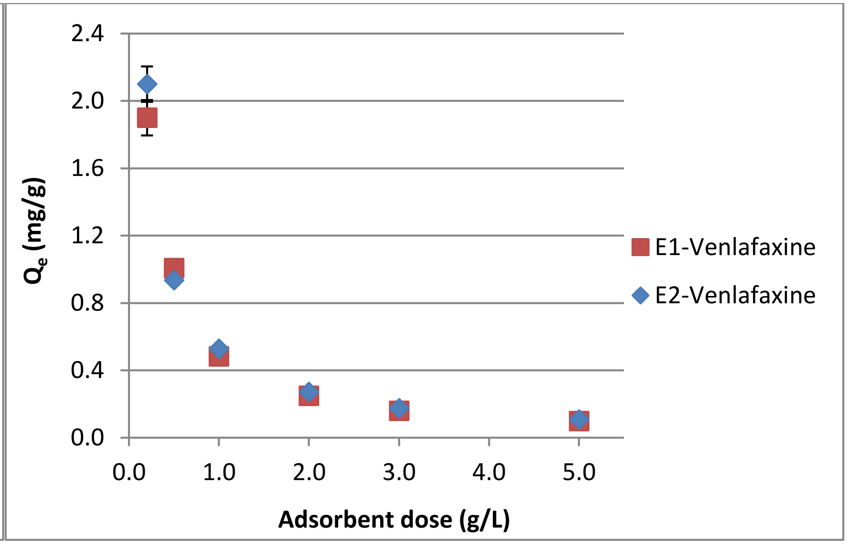

(j)

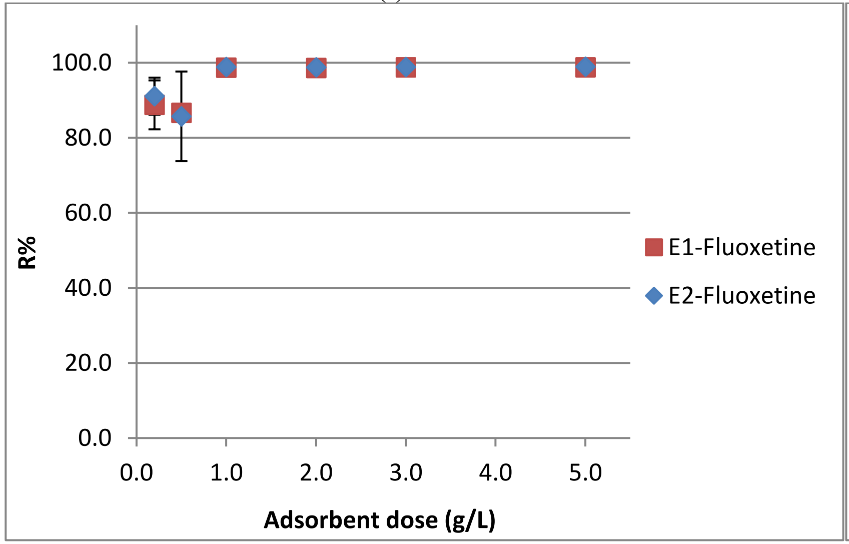

(k)

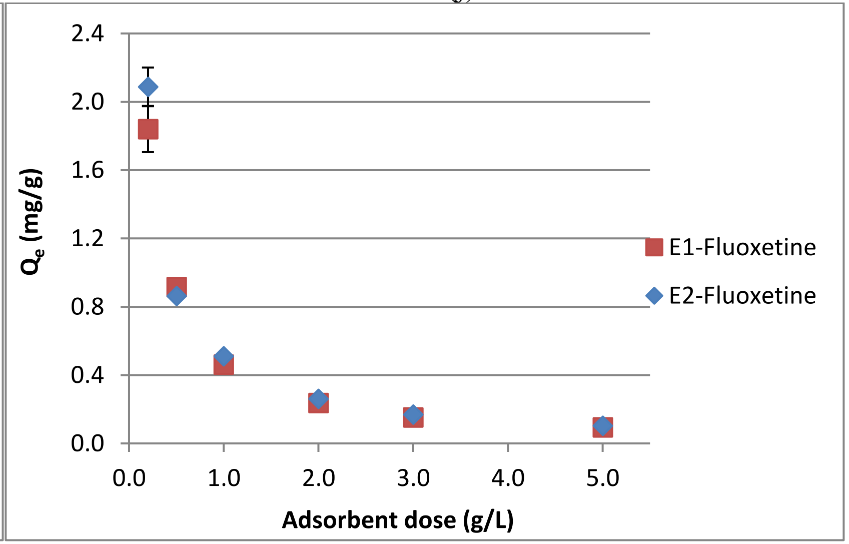

(l)

Figure S5 (continued). Effects of adsorbent dose (i) %R for venlafaxine (j) Q_e for venlafaxine (k) %R for fluoxetine (l) Q_e for fluoxetine

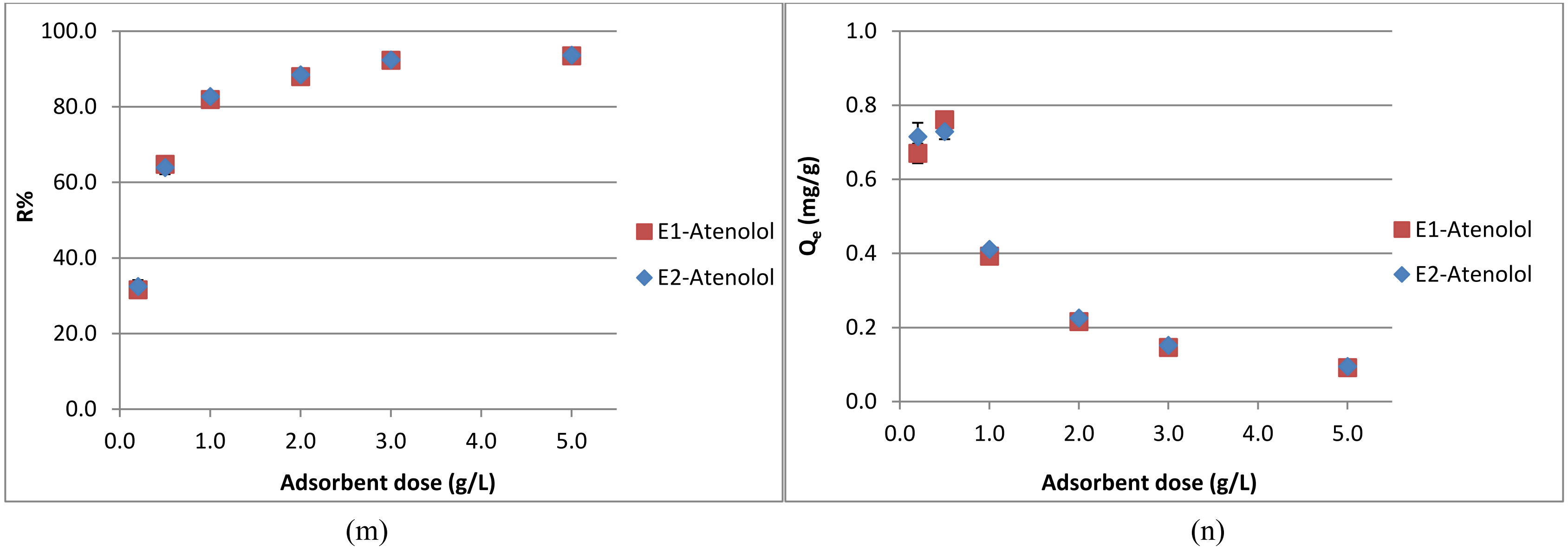

Figure S5 (continued). Effects of adsorbent dose (m) %R for atenolol (n) Q_e for atenolol

Table S1. Paired sample t-test results for percent removal and Q_e for E1 and E2 (α= 0.01, df= 11, t_Critical= 3.106)

	**Metoprolol**	**Propranolol**	**Venlafaxine**	**Fluoxetine**	**Atenolol**	**O-desmethylvenlafaxine**
**t Stat for %R (E1 and E2)**	1.066	-1.987	1.205	-0.217	-1.334	-0.595
**t Stat for Q_e (E1 and E2)**	0.546	-0.278	-1.500	-1.182	-1.151	-2.375

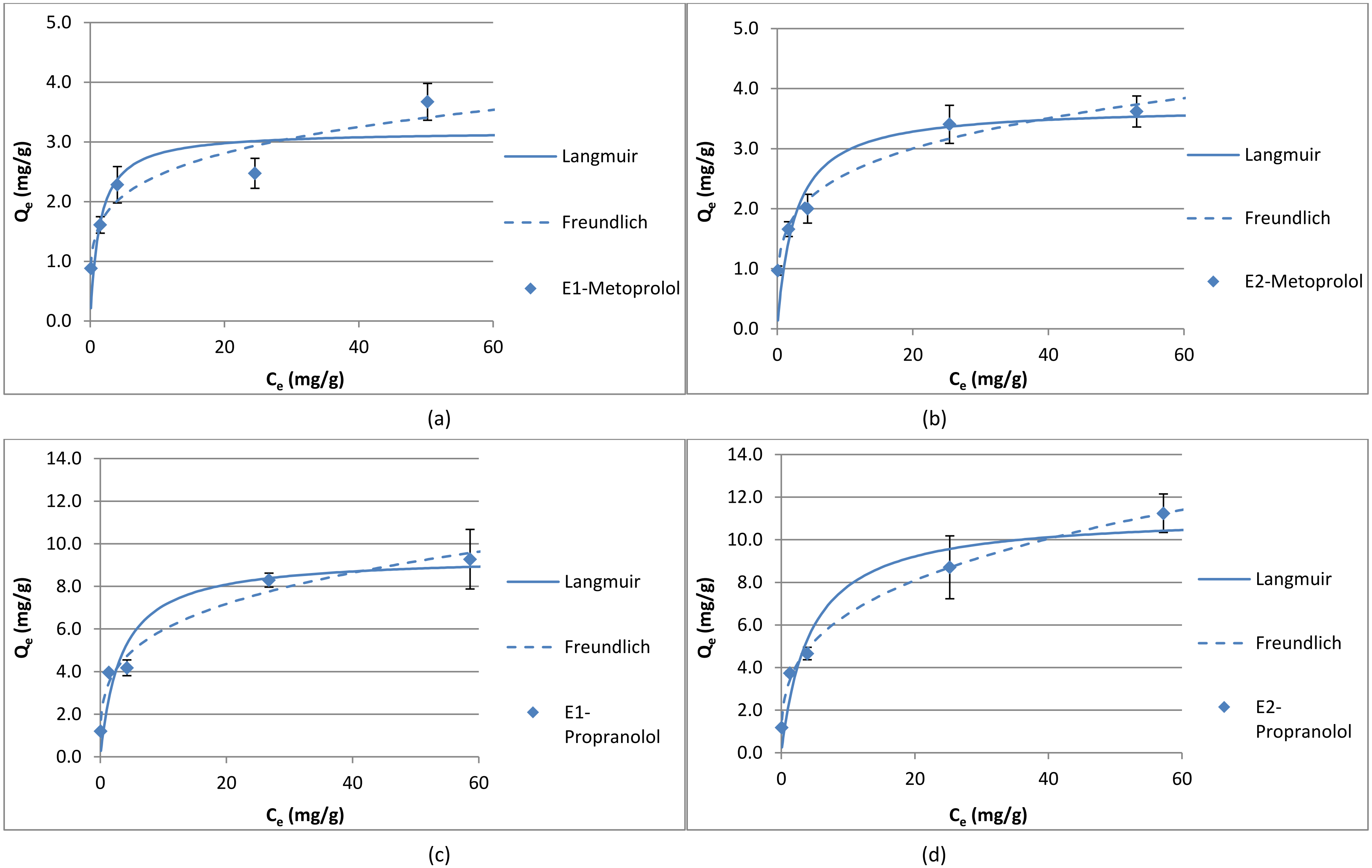

Figure S6. Equilibrium data and isotherm model fit (a) E1-Metoprolol (b) E2-Metoprolol (c) E1-Propranolol (d) E2-Propranolol

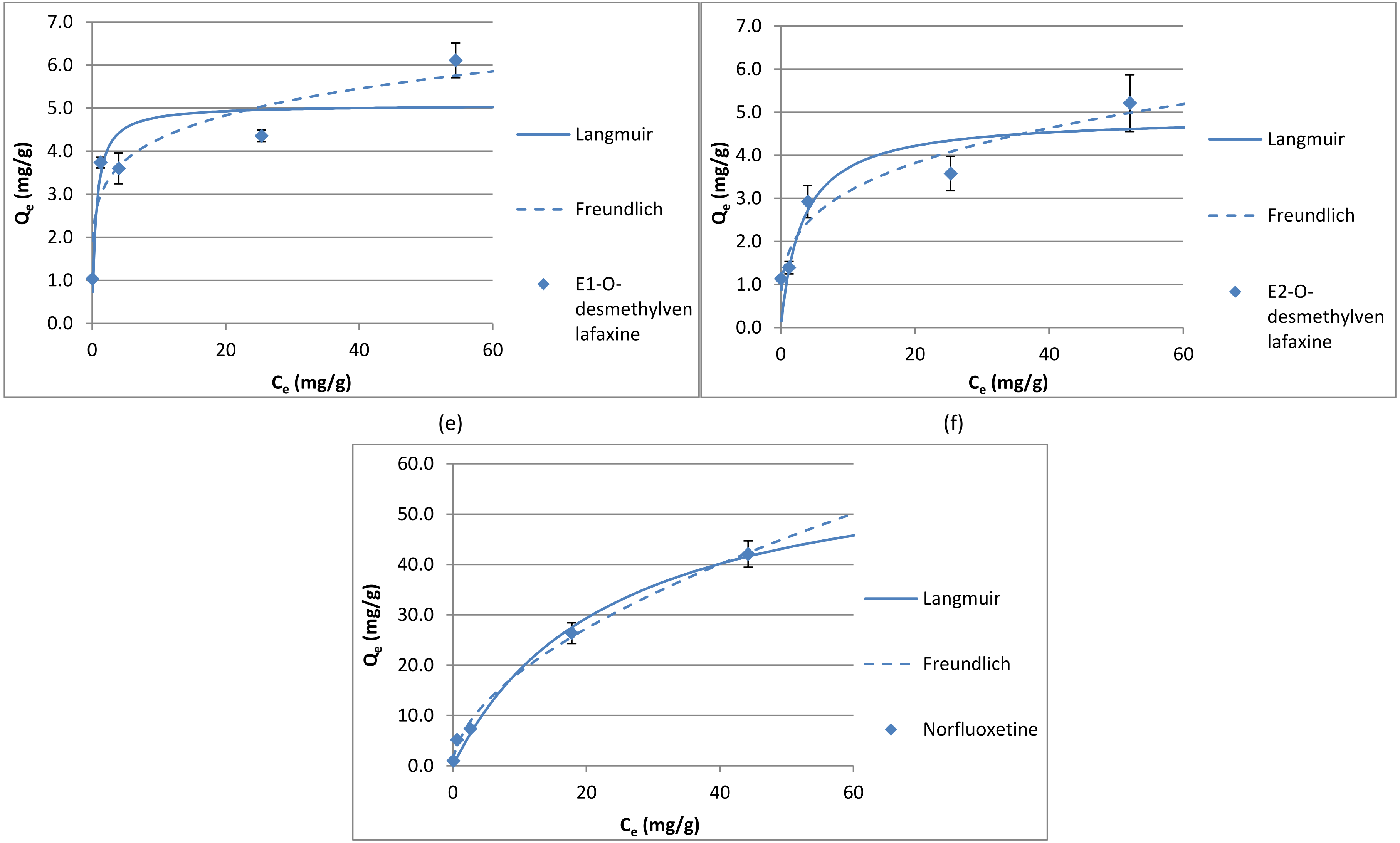

(e) (f) (g)

Figure S6 (cont). Equilibrium data and isotherm model fit (e) E1-O-desmethylvenlafaxine (f) E2-O-desmethylvenlafaxine (g) Norfluoxetine

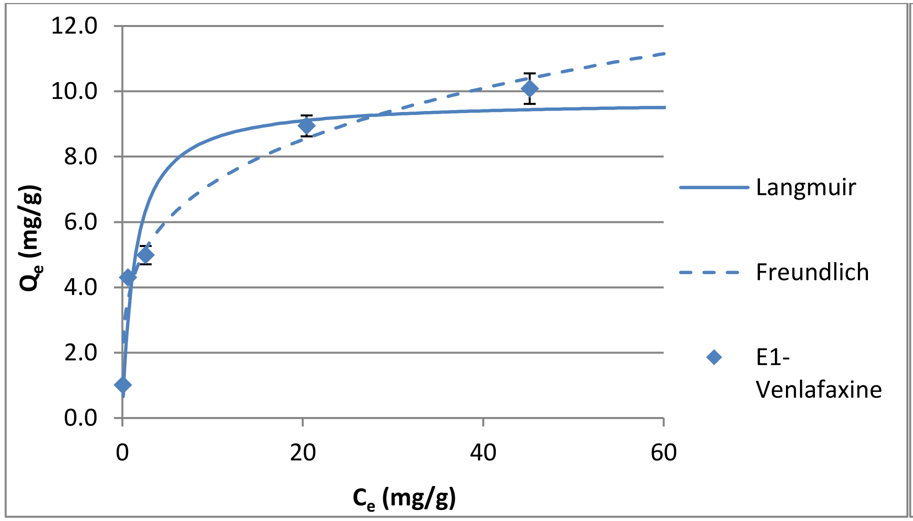

(h)

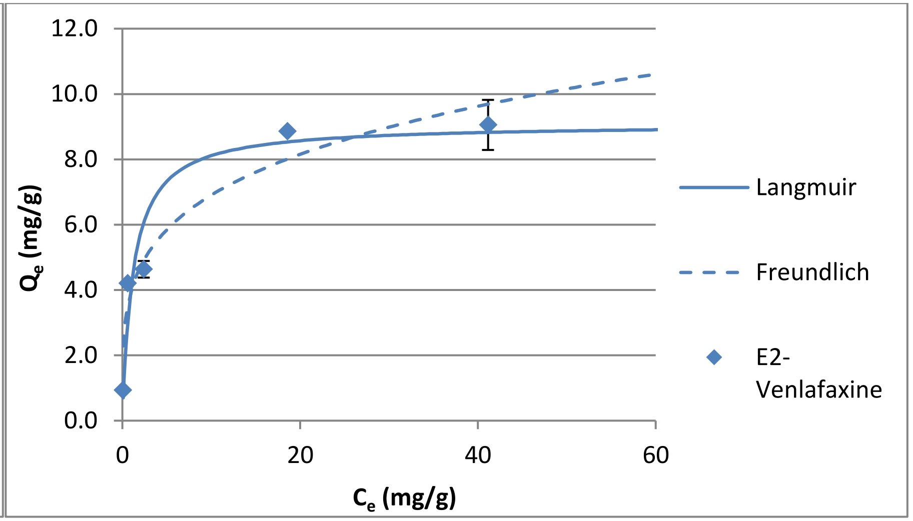

(i)

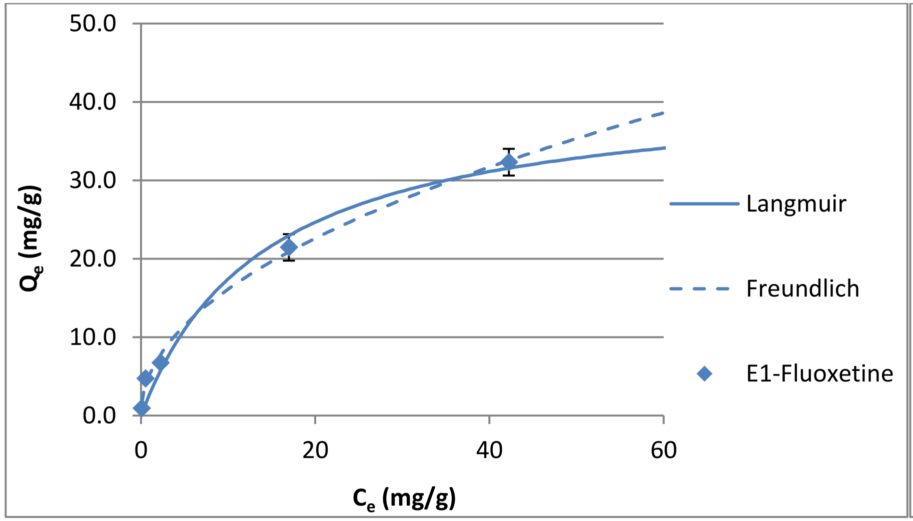

(j)

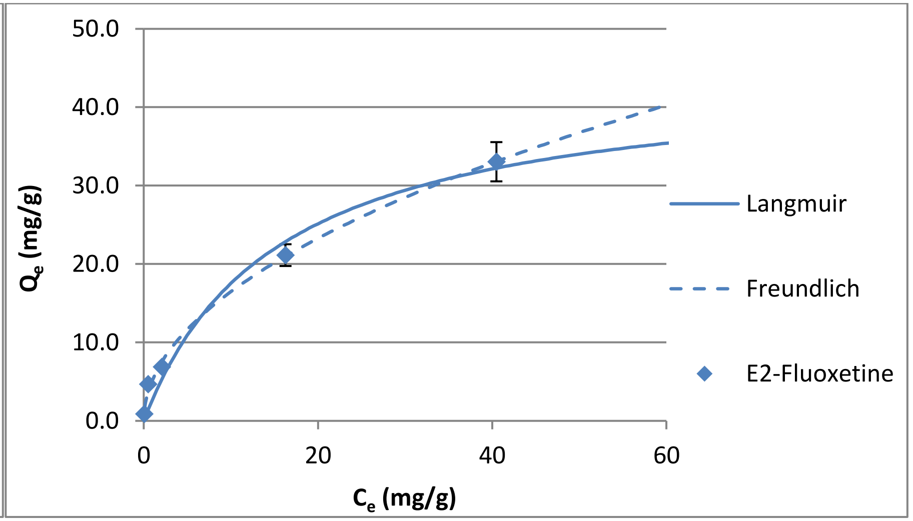

(k)

Figure S6 (cont). Equilibrium data and isotherm model fit (h) E1-Venlafaxine (i) E2-Venlafaxine (j) E1-Fluoxetine (k) E2-Fluoxetine

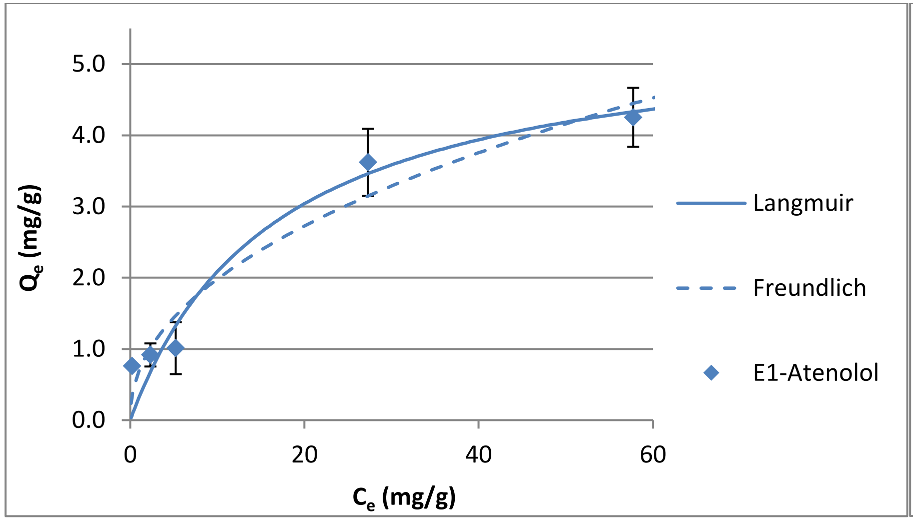

(l)

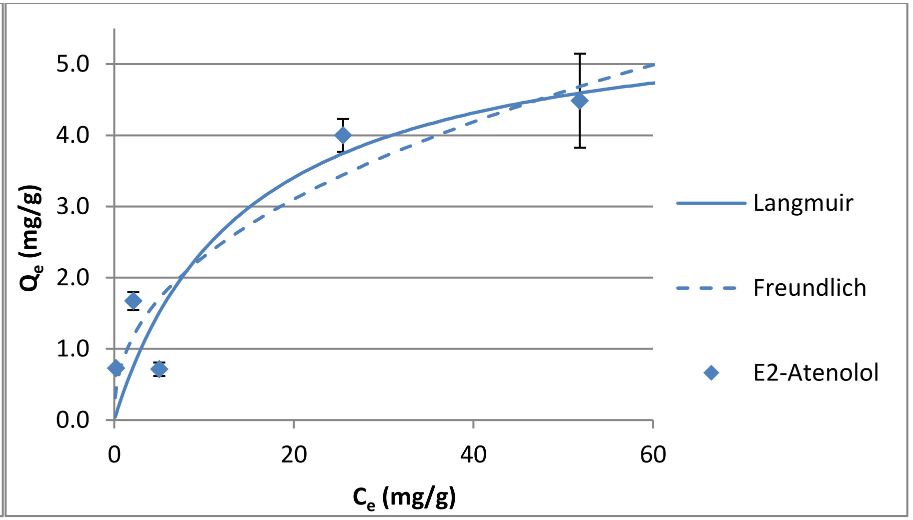

(m)

Figure S6 (cont). Equilibrium data and isotherm model fit (l) E1-Atenolol (m) E2-Atenolol

Table S2. Isotherm model fit parameters

Adsorbate	Model*	Model Parameters		R^2	χ^2	NRMSE
**E1-Metoprolol**	**Langmuir**	Q_{max}	3.2	0.750	2.427	0.167
		K_L	0.724			
	**Freundlich**	K_F	1.505	0.918	0.131	0.096
		N	0.209			
**E2-Metoprolol**	**Langmuir**	Q_{max}	3.7	0.827	6.172	0.161
		K_L	0.393			
	**Freundlich**	K_F	1.531	0.980	0.042	0.054
		n	0.225			
**E1-Propranolol**	**Langmuir**	Q_{max}	9.4	0.911	8.467	0.111
		K_L	0.305			
	**Freundlich**	K_F	3.218	0.978	0.219	0.055
		n	0.268			
**E2-Propranolol**	**Langmuir**	Q_{max}	11.2	0.936	8.052	0.091
		K_L	0.230			
	**Freundlich**	K_F	3.176	0.998	0.041	0.016
		n	0.312			
**E1-O-desmethyl venlafaxine**	**Langmuir**	Q_{max}	5.1	0.777	3.706	0.152
		K_L	1.703			
	**Freundlich**	K_F	2.865	0.896	0.454	0.104
		n	0.174			

Table S2 (continued). Isotherm model fit parameters

**E2-O-desmethyl venlafaxine**	**Langmuir**	Q_{max}	4.9	0.822	7.262	0.155
		K_L	0.310			
	**Freundlich**	K_F	1.661	0.928	0.362	0.099
		n	0.278			
**Norfluoxetine**	**Langmuir**	Q_{max}	63.6	0.987	12.045	0.043
		K_L	0.043			
	**Freundlich**	K_F	5.188	0.996	0.633	0.023
		n	0.554			
**E1-Venlafaxine**	**Langmuir**	Q_{max}	9.7	0.915	3.139	0.106
		K_L	0.729			
	**Freundlich**	K_F	4.092	0.974	0.529	0.059
		n	0.245			
**E2-Venlafaxine**	**Langmuir**	Q_{max}	9.1	0.914	2.002	0.111
		K_L	0.835			
	**Freundlich**	K_F	3.974	0.945	0.770	0.089
		n	0.240			
**E1-Fluoxetine**	**Langmuir**	Q_{max}	42.2	0.978	10.538	0.056
		K_L	0.070			
	**Freundlich**	K_F	5.250	0.996	0.594	0.023
		n	0.487			

Table S2 (continued). Isotherm model fit parameters

**E2-Fluoxetine**	**Langmuir**	Q_{max}	44.5	0.976	10.728	0.058
		K_L	0.065			
	**Freundlich**	K_F	5.221	0.998	0.528	0.018
		n	0.499			
**E1-Atenolol**	**Langmuir**	Q_{max}	5.6	0.940	7.542	0.105
		K_L	0.060			
	**Freundlich**	K_F	0.685	0.941	0.812	0.105
		n	0.461			
**E2-Atenolol**	**Langmuir**	Q_{max}	5.9	0.848	6.936	0.167
		K_L	0.069			
	**Freundlich**	K_F	0.848	0.872	1.110	0.153
		n	0.433			

* The isotherm models used are presented below:

Langmuir: $q_e = \frac{Q_{max}K_LC_e}{1+ K_LC_e}$ (S1)

Freundlich: $q_e = K_FC_e^n$ (S2)

where, q_e (mg/g) is the amount of pollutant uptake at equilibrium; C_e (mg/L) is the equilibrium concentration of pollutant; Q_{max} (mg/g) is the maximum adsorption capacity calculated from Langmuir model; K_L (L/mg) is the Langmuir constant; K_F ((mg/g)/(mg/L)^n) is the Freundlich constant; n (dimensionless) is the Freundlich intensity parameter.

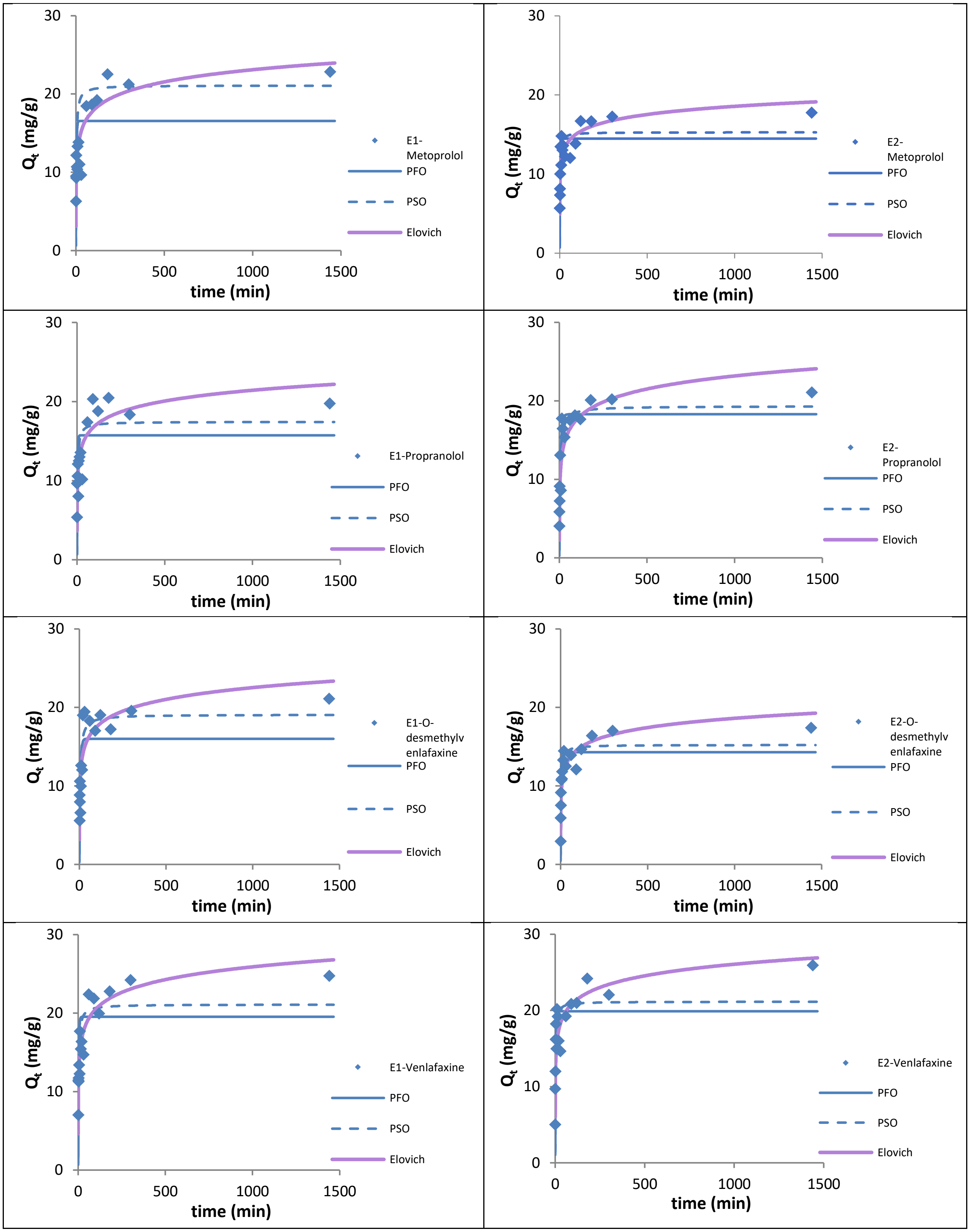

Figure S7. Kinetic results and model fit for E1-Metoprolol, E2-Metoprolol, E1-Propranolol, E2, Propranolol, E1-Venlafaxine, E2-Venlafaxine, E1-O-desmethylvenlafaxine, E2-O-desmethylvenlafaxine (error bars are not shown for clarity; RSD<5%)

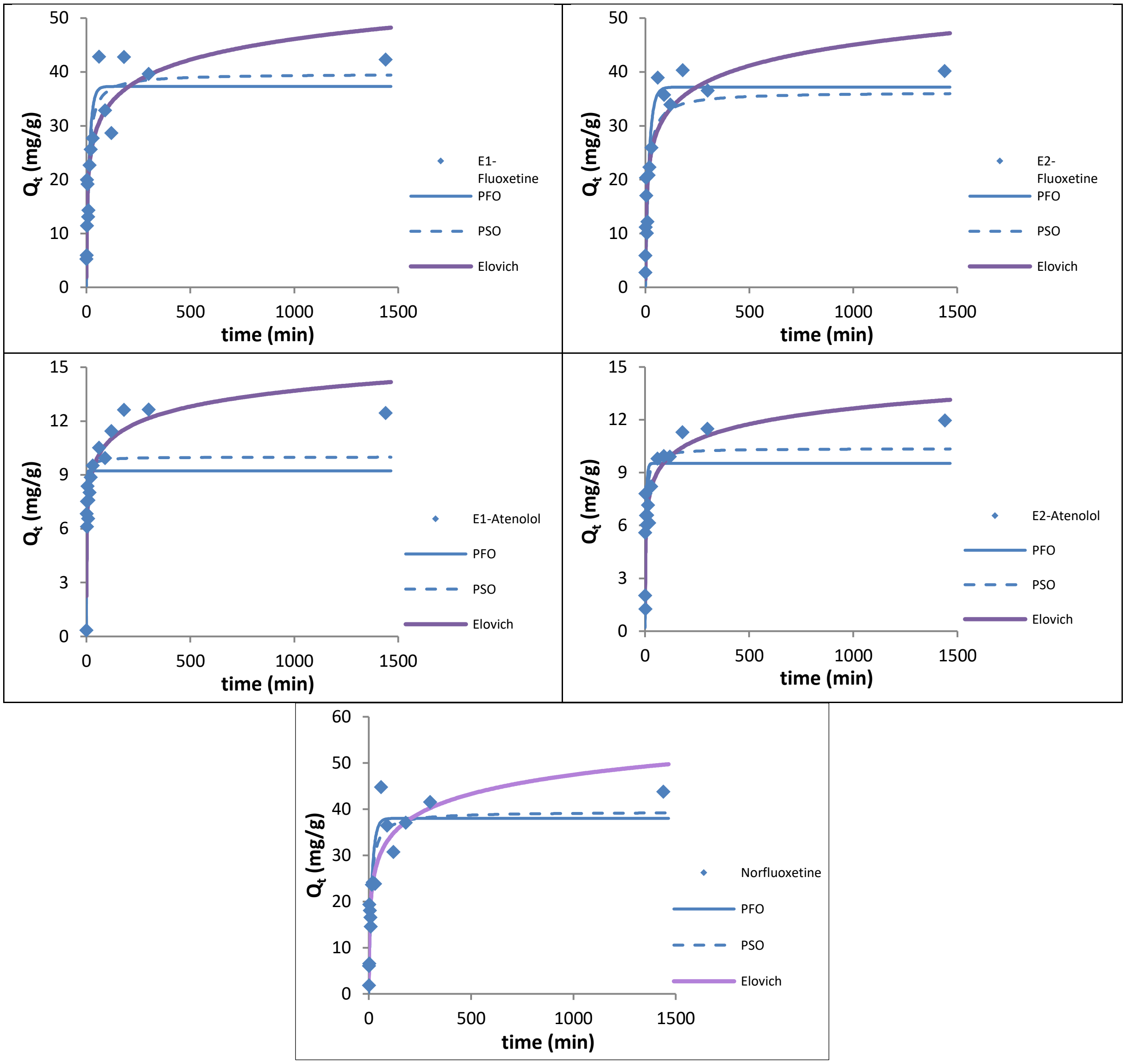

Figure S7 (continued). Kinetic results and model fit for E1-Fluoxetine, E2-Fluoxetine, E1-Atenolol, E2-Atenolol, Norfluoxetine

Table S3. Kinetic model fit parameters

Compound	Model*	Model Parameters	R^2	χ^2	NRMSE
**E1-Metoprolol**	**PFO**	k_1 (min^{-1}) = 0.394<br>q_e (mg/g) = 16.559	0.252	22.239	0.351
	**PSO**	k_2 (g/(mg×min)) = 0.019<br>q_e (mg/g) = 18.613	0.207	18.376	0.559
	**Elovich**	α (mg/(g×min)) = 66.711<br>β (g/mg) = 0.446	0.127	4.927	0.834
**E2-Metoprolol**	**PFO**	k_1 (min^{-1}) = 0.503<br>q_e (mg/g) = 14.482	0.166	6.264	0.672
	**PSO**	k_2 (g/(mg×min)) = 0.049<br>q_e (mg/g) = 15.286	0.138	3.561	0.771
	**Elovich**	α (mg/(g×min)) = 401.598<br>β (g/mg) = 0.674	0.124	3.062	0.817
**E1-Propranolol**	**PFO**	k_1 (min^{-1}) = 0.436<br>q_e (mg/g) = 15.732	0.240	16.775	0.398
	**PSO**	k_2 (g/(mg×min)) = 0.025<br>q_e (mg/g) = 17.442	0.194	12.754	0.606
	**Elovich**	α (mg/(g×min)) = 106.260<br>β (g/mg) = 0.509	0.139	5.039	0.798
**E2-Propranolol**	**PFO**	k_1 (min^{-1}) = 0.145<br>q_e (mg/g) = 18.297	0.159	19.328	0.758
	**PSO**	k_2 (g/(mg×min)) = 0.012<br>q_e (mg/g) = 19.324	0.128	8.289	0.843
	**Elovich**	α (mg/(g×min)) = 37.809<br>β (g/mg) = 0.147	0.125	5.719	0.850
**E1-O-desmethyl venlafaxine**	**PFO**	k_1 (min^{-1}) = 0.185<br>q_e (mg/g) = 16.015	0.218	37.144	0.567
	**PSO**	k_2 (g/(mg×min)) = 0.014<br>q_e (mg/g) = 19.085	0.164	15.766	0.753
	**Elovich**	α (mg/(g×min)) = 72.165<br>β (g/mg) = 0.462	0.150	6.482	0.794

Table S3 (continued). Kinetic model fit parameters

**E2-O-desmethyl venlafaxine**	**PFO**	k_1 (min^{-1}) = 0.322<br>q_e (mg/g) = 14.296	0.116	4.280	0.813
	**PSO**	k_2 (g/(mg×min)) = 0.031<br>q_e (mg/g) = 15.225	0.087	1.898	0.896
	**Elovich**	α (mg/(g×min)) = 93.951<br>β (g/mg) = 0.586	0.097	3.256	0.870
**Norfluoxetine**	**PFO**	k_1 (min^{-1}) = 0.066<br>q_e (mg/g) = 38.001	0.130	42.742	0.824
	**PSO**	k_2 (g/(mg×min)) = 0.003<br>q_e (mg/g) = 39.425	0.111	20.415	0.873
	**Elovich**	α (mg/(g×min)) = 16.461<br>β (g/mg) = 0.167	0.114	17.676	0.864
**E1-Venlafaxine**	**PFO**	k_1 (min^{-1}) = 0.347<br>q_e (mg/g) = 19.531	0.209	19.672	0.502
	**PSO**	k_2 (g/(mg×min)) = 0.022<br>q_e (mg/g) = 21.096	0.160	11.119	0.705
	**Elovich**	α (mg/(g×min)) = 145.175<br>β (g/mg) = 0.426	0.095	2.736	0.898
**E2-Venlafaxine**	**PFO**	k_1 (min^{-1}) = 0.528<br>q_e (mg/g) = 19.893	0.136	6.764	0.704
	**PSO**	k_2 (g/(mg×min)) = 0.034<br>q_e (mg/g) = 21.155	0.120	5.162	0.771
	**Elovich**	α (mg/(g×min)) = 317.810<br>β (g/mg) = 0.456	0.114	6.480	0.794
**E1-Fluoxetine**	**PFO**	k_1 (min^{-1}) = 0.072<br>q_e (mg/g) = 37.3	0.157	61.068	0.776
	**PSO**	k_2 (g/(mg×min)) = 0.003<br>q_e (mg/g) = 39.66	0.138	29.516	0.827
	**Elovich**	α (mg/(g×min)) = 22.6<br>β (g/mg) = 0.18	0.131	15.606	0.844
**E2-Fluoxetine**	**PFO**	k_1 (min^{-1}) = 0.057<br>q_e (mg/g) = 37.2	0.139	69.252	0.824
	**PSO**	k_2 (g/(mg×min)) = 0.002<br>q_e (mg/g) = 36.25	0.140	43.679	0.825
	**Elovich**	α (mg/(g×min)) = 17.1<br>β (g/mg) = 0.18	0.128	17.833	0.851

Table S3 (continued). Kinetic model fit parameters

**E1-Atenolol**	**PFO**	k_1 (min^{-1}) = 0.636<br>q_e (mg/g) = 9.225	0.170	9.789	0.487
	**PSO**	k_2 (g/(mg×min)) = 0.069<br>q_e (mg/g) = 9.997	0.154	8.699	0.583
	**Elovich**	α (mg/(g×min)) = 61.910<br>β (g/mg) = 0.789	0.110	5.511	0.801
**E2-Atenolol**	**PFO**	k_1 (min^{-1}) = 0.210<br>q_e (mg/g) = 9.53	0.190	16.912	0.545
	**PSO**	k_2 (g/(mg×min)) = 0.026<br>q_e (mg/g) = 10.37	0.157	11.196	0.692
	**Elovich**	α (mg/(g×min)) = 24.19<br>(g/mg) = 0.78	0.125	5.614	0.805

* The kinetic models used are presented below:

Pseudo-first order (PFO) : $q_t = q_e\left(1 - e^{-k_1 t}\right)$ (S3)

Pseudo-second order (PSO): $q_t = \frac{q_e^2 k_2 t}{1+q_e k_2 t}$ (S4)

Elovich: $q_t = \frac{1}{\beta}\ln(1 + \alpha\beta t)$ (S5)

where, q_t and q_e are the amounts of pollutant adsorbed (mg/g) at time, t (min) and at equilibrium; k_1 is the rate constant of pseudo-first order equation (1/min); k_2 is the rate constant of pseudo-second order equation (g/(mg·min)); α and β are constants.

Table S4. Physical and chemical properties of the target pharmaceuticals used

**Compound name**	**CAS No.**	**Chemical Formula**	**Molecular Weight (g/mol)**	**pK_a**	**Ref. No.**
**Atenolol**	29122-68-7	$C_{14}H_{22}N_2O_3$	266.34	9.6	[32]
**Fluoxetine**	54910-89-3	$C_{17}H_{18}F_3NO$	309.33	9.8	[32]
**Metoprolol**	37350-58-6	$C_{15}H_{25}NO_3$	267.36	9.7	[32]
**Norfluoxetine**	83891-03-6	$C_{16}H_{16}F_3NO$	295.30	9.4	[33]
**O-desmethylvenlafaxine**	142761-12-4	$C_{16}H_{25}NO_2$	263.37	8.9	[32]
**Propranolol**	525-66-6	$C_{16}H_{21}NO_2$	259.34	9.4	[32]
**Venlafaxine**	93413-69-5	$C_{17}H_{27}NO_2$	277.40	8.9	[32]

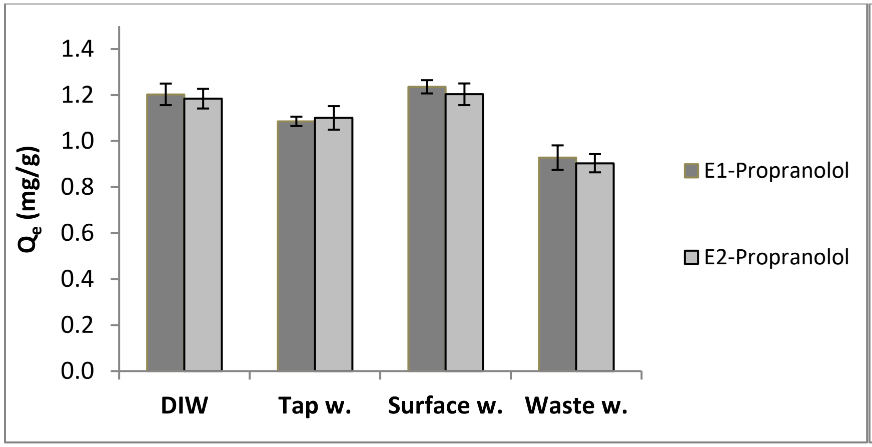

(a)

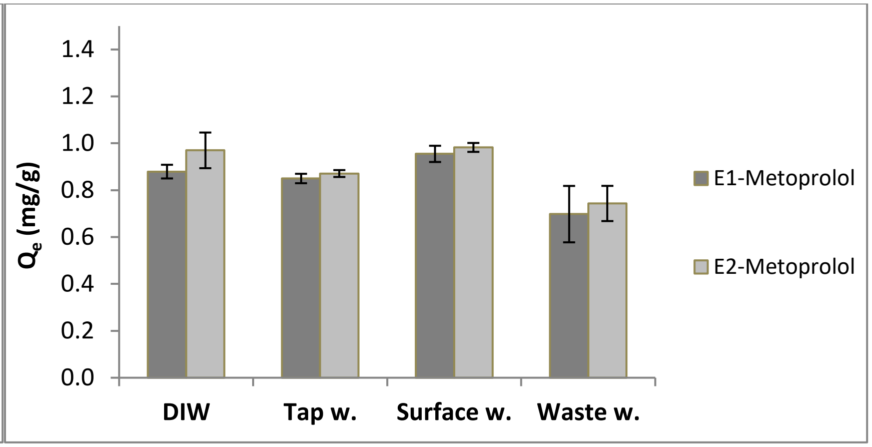

(b)

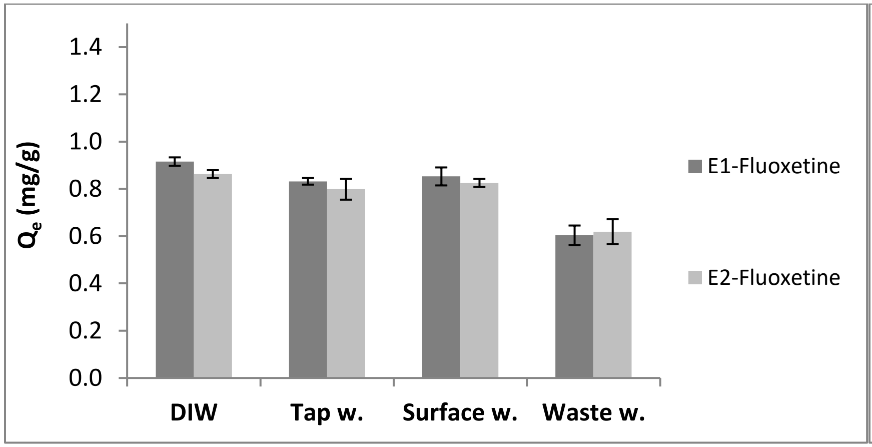

(c)

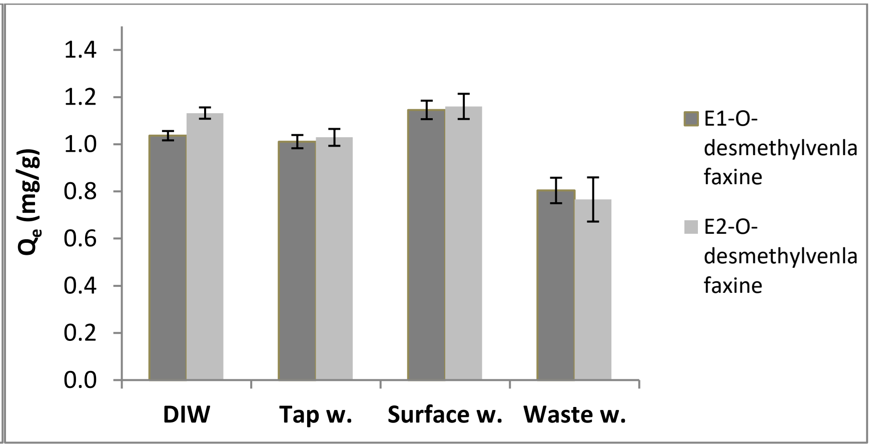

(d)

Figure S8. Effects of deionized water (DIW), tap water, surface water and wastewater (a) Propranolol (b) Metoprolol (c) Fluoxetine (d) O-desmethylvenlafaxine

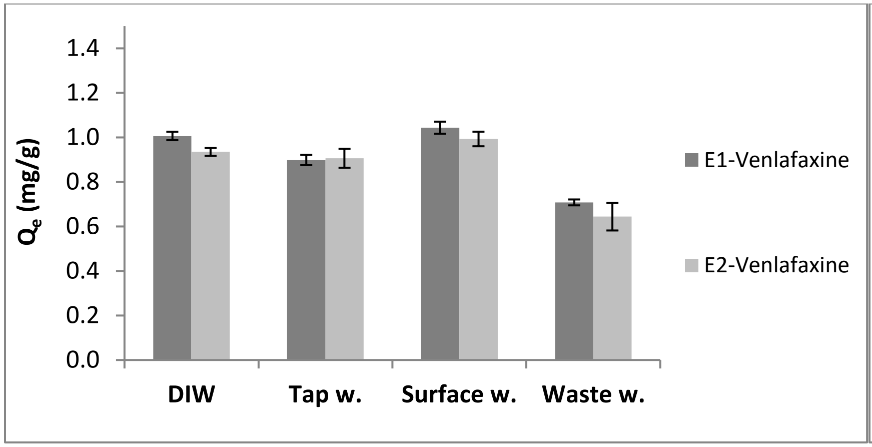

(e)

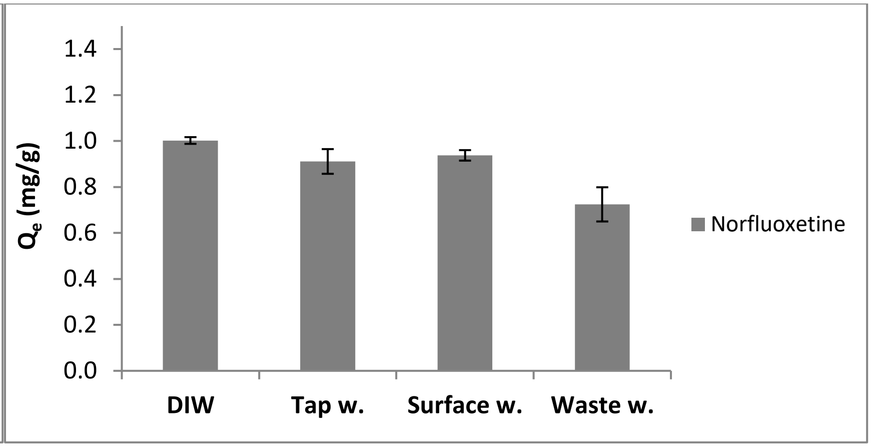

(f)

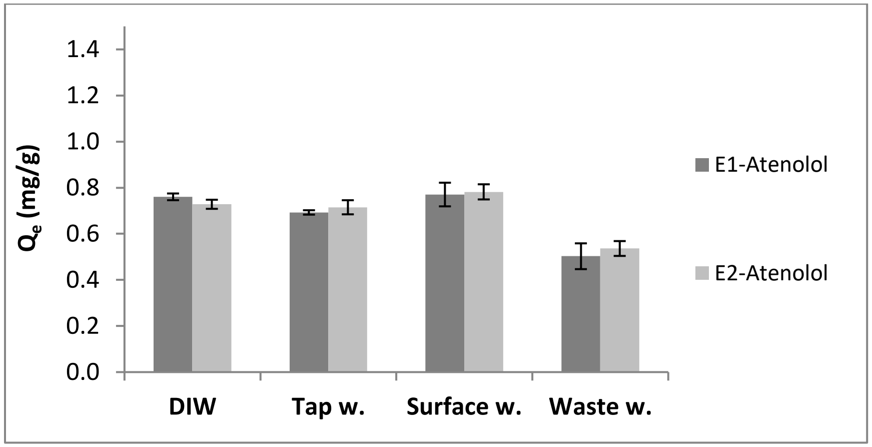

(g)

Figure S8 (continued). Effects of deionized water (DIW), tap water, surface water and wastewater (e) Venlafaxine (f) Norfluoxetine (g) Atenolol

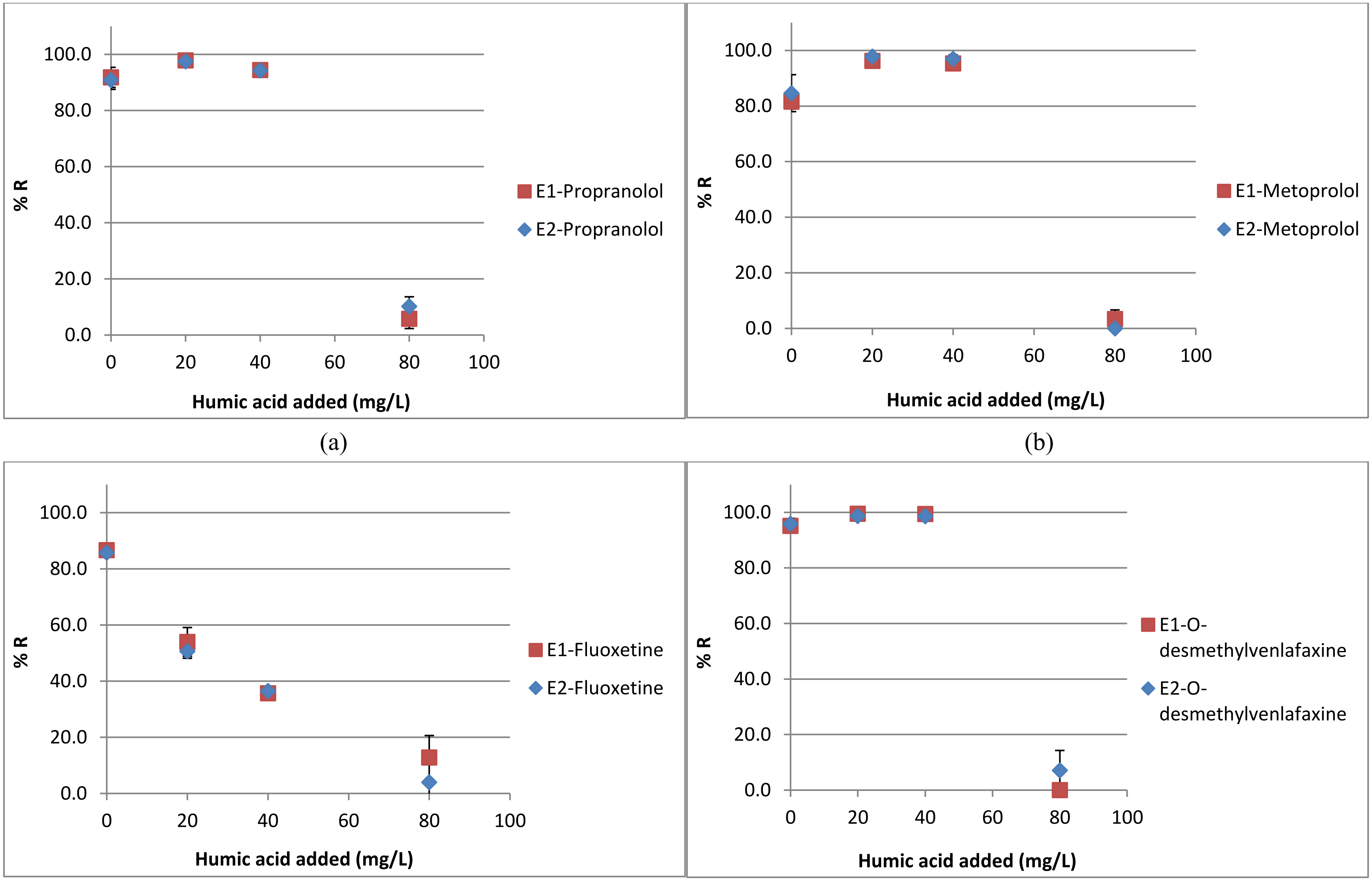

(a) (b)

(c) (d)

Figure S9. Effects of humic acid on the adsorption of (a) Propranolol (b) Metoprolol (c) Fluoxetine (d) O-desmethylvenlafaxine

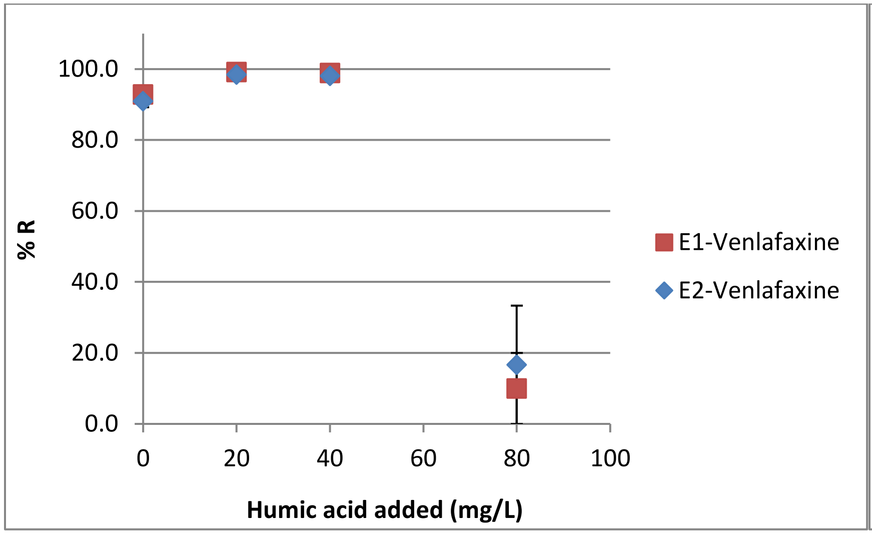

(e)

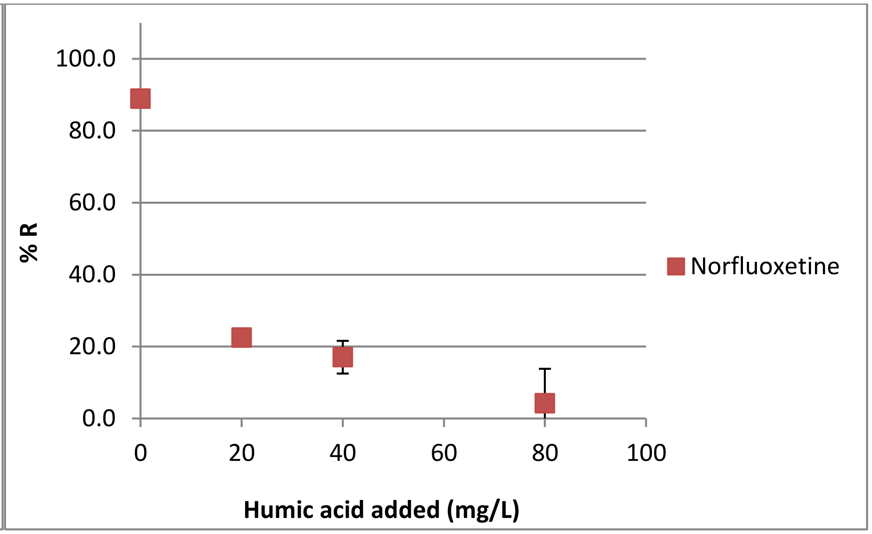

(f)

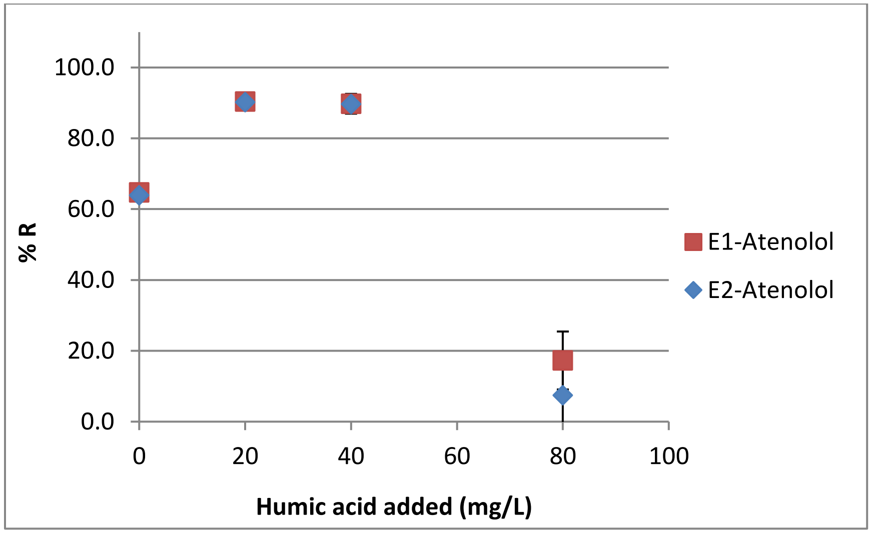

(g)

Figure S9 (continued). Effects of humic acid on the adsorption of (e) Venlafaxine (f) Norfluoxetine (g) Atenolol

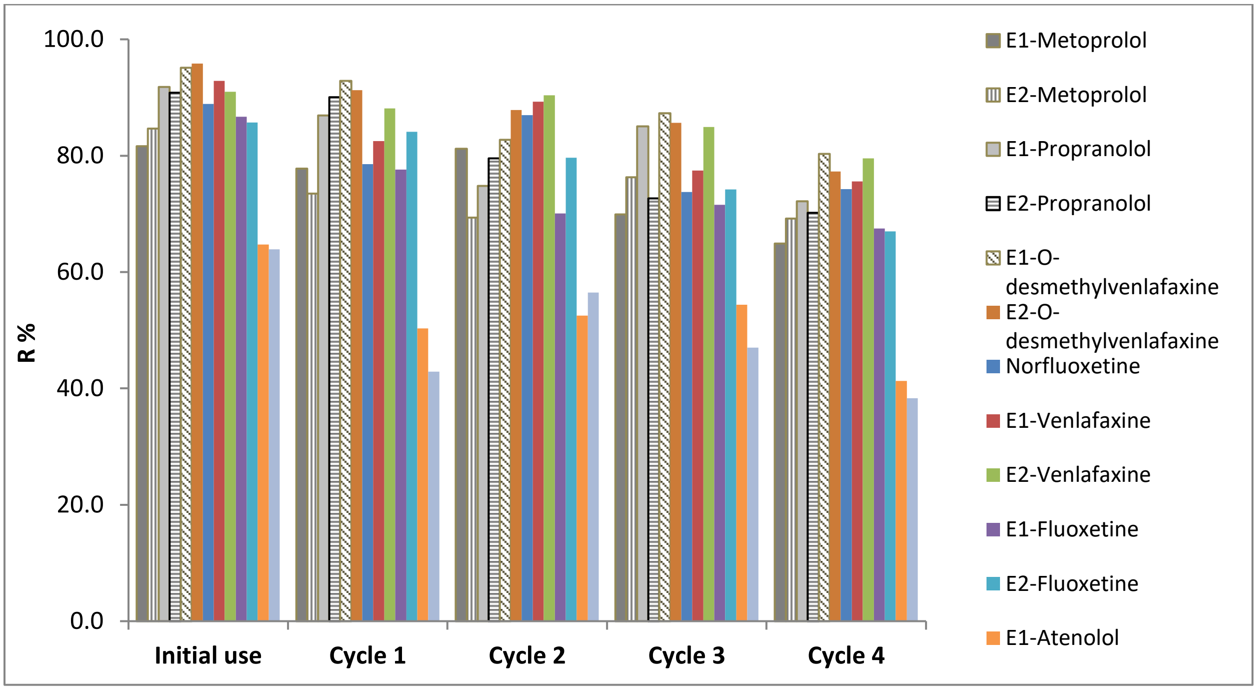

Figure S10. Four-cycle desorption and reuse results
(error bars are not shown for clarity; RSD<5%)

Table S5. Experimental conditions used in adsorption studies*

Experiment type	Contact time (min)	Adsorbent dose (g/L)	Solution volume (ml)	Initial adsorbate concentration (mg/L)**	Adjusted pH	HA added (mg/L)
Effects of adsorbent dose	1440	0.2-5	10	0.5	no pH adjustment	0
Adsorption kinetics	0.5-1440	0.5	200	5	no pH adjustment	0
Adsorption isotherms	1440	0.5	10	0.5-50	no pH adjustment	0
Effects of pH	1440	0.5	10	0.5	2, 4, 6, 8	0
Effects of humic acid	1440	0.5	10	0.5	no pH adjustment	0- 80
Experiments with tap water, surface water and wastewater	1440	0.5	10	0.5	no pH adjustment	0
Desorption and reuse	1440	0.5	10	0.5	no pH adjustment	0

* The constant parameters were mixing speed (350 rpm) and temperature (25 ± 1 ºC).

** Given for each enantiomer in the mixture.

Table S6. LC–MS/MS method parameters and coefficient of determination (R^2) of calibration

Compound	Ionization mode	Precursor ion (m/z)	Product ions (quantifier/qualifier) (m/z)	Fragmentor (V)	Collision energy (V)	Retention time (min)	R^2
E1-Atenolol	Positive	267.4	266.9/145.3	166	28/36	14.04	0.998
E2-Atenolol						15.48	0.995
E1-Fluoxetine	Positive	310.1	44.0/148.1	166	12/4	12.62	0.999
E2-Fluoxetine						14.04	0.995
E1-Metoprolol	Positive	268.4	133.0/103.1	166	28/44	9.81	0.995
E2-Metoprolol						10.87	0.997
Norfluoxetine	Positive	296.1	134.1/105.0	166	0/24	11.65	0.994
E1-O-desmethyl venlafaxine	Positive	264.4	264.0/246.0	166	4/12	10.87	0.999
E1-O-desmethyl venlafaxine						11.51	0.992
E1-Propranolol	Positive	260.4	259.9/116.1	166	0/20	10.94	0.999
E1-Propranolol						11.8	0.969
E1-Venlafaxine	Positive	278.2	260.2/121.0	166	12/36	11.64	0.999
E1-Venlafaxine						13.02	0.998

Table S7. Parameters used for model evaluation

Parameter	Equation
Coefficient of determination	$R^2 = 1 - \frac{\sum_{i=1}^{n}(q_{exp} - q_{calc})^2}{\sum_{i=1}^{n}(q_{exp} - q_{exp,mean})^2}$
Chi square	$\chi^2 = \sum_{i=1}^{n}\frac{(q_{exp} - q_{calc})^2}{q_{calc}}$
Normalized root mean square error	$\mathrm{NRMSE} = \frac{\sqrt{\frac{\sum_{i=1}^{n}(q_{exp} - q_{calc})^2}{n}}}{q_{exp,max} - q_{exp,min}}$

Where n is the number of data points; q_{exp} and q_{calc} are the quantities adsorbed determined experimentally and the quantities adsorbed calculated by the models, respectively; $q_{exp,max}$ and $q_{exp,min}$, $q_{exp,mean}$ are the maximum, minimum and mean values of the quantities adsorbed determined experimentally, respectively.
